# Clinical characteristics and predictors of outcomes of hospitalized patients with COVID-19 in a multi-ethnic London NHS Trust: a retrospective cohort study

**DOI:** 10.1093/cid/ciaa1091

**Published:** 2020-08-07

**Authors:** Pablo N Perez-Guzman, Anna Daunt, Sujit Mukherjee, Peter Crook, Roberta Forlano, Mara D Kont, Alessandra Løchen, Michaela Vollmer, Paul Middleton, Rebekah Judge, Christopher Harlow, Anet Soubieres, Graham Cooke, Peter J White, Timothy B Hallett, Paul Aylin, Neil Ferguson, Katharina Hauck, Mark R Thursz, Shevanthi Nayagam

**Affiliations:** 1 MRC Centre for Global Infectious Disease Analysis and Abdul Latif Jameel Institute for Disease and Emergency Analytics (J-IDEA), School of Public Health, Imperial College London, United Kingdom; 2 Division of Digestive Diseases, Department of Metabolism, Digestion and Reproduction, Faculty of Medicine, Imperial College London, United Kingdom; 3 Imperial College Healthcare NHS Trust, London, United Kingdom; 4 Department of Infectious Diseases, Imperial College London, United Kingdom; 5 Department of Primary Care and Public Health, School of Public Health, Imperial College London, United Kingdom

**Keywords:** COVID-19, mortality, ethnic minority groups

## Abstract

**Background:**

Emerging evidence suggests ethnic minorities are disproportionately affected by COVID-19. Detailed clinical analyses of multi-cultural hospitalized patient cohorts remain largely undescribed.

**Methods:**

We performed regression, survival and cumulative competing risk analyses to evaluate factors associated with mortality in patients admitted for COVID-19 in three large London hospitals between February 25 and April 5, censored as of May 1, 2020.

**Results:**

Of 614 patients (median age 69 years, (IQR 25) and 62% male), 381 (62%) had been discharged alive, 178 (29%) died and 55 (9%) remained hospitalized at censoring. Severe hypoxemia (aOR 4.25, 95%CI 2.36-7.64), leukocytosis (aOR 2.35, 95%CI 1.35-4.11), thrombocytopenia (aOR 1.01, 95%CI 1.00-1.01, increase per 10x^9^ decrease), severe renal impairment (aOR 5.14, 95%CI 2.65-9.97), and low albumin (aOR 1.06, 95%CI 1.02-1.09, increase per g decrease) were associated with death. Forty percent (244) were from black, Asian and other minority ethnic (BAME) groups, 38% (235) white and for 22% (135) ethnicity was unknown. BAME patients were younger and had fewer comorbidities. Whilst the unadjusted odds of death did not differ by ethnicity, when adjusting for age, sex and comorbidities, black patients were at higher odds of death compared to whites (aOR 1.69, 95%CI 1.00-2.86). This association was stronger when further adjusting for admission severity (aOR 1.85 95% CI 1.06-3.24).

**Conclusions:**

BAME patients were over-represented in our cohort and, when accounting for demographic and clinical profile of admission, black patients were at increased odds of death. Further research is needed into biologic drivers of differences in COVID-19 outcomes by ethnicity.

## 1. Introduction

The United Kingdom (UK) has been the 3rd worst affected country in the world by COVID-19, with a reported death toll of 42,2927 as of the 23rd of June 2020.[1] Most deaths have occurred in London,[2] a densely populated, multicultural capital city where over 40% of residents identify themselves as belonging to a black, Asian or other ethnic minority (BAME).[3]

Previous studies have found that age, gender, and co-morbidities including cardiovascular disease are associated with poorer COVID-19 hospitalization outcomes.[4–8] However, robust analyses adjusting for confounding factors and evaluating the association of the physiological and laboratory characteristics on admission with clinical outcomes are lacking. Such studies are urgently needed to allow frontline clinicians to tailor their management decisions and hospital policymakers to efficiently plan their response to COVID-19.

Data from UK admission to intensive care units (ICU) and from the Office for National Statistics (ONS) suggests BAME groups are more frequently affected with COVID-19 than white patients.[9,10] This is largely thought to be due to economic deprivation driving an increased risk of COVID-19 infection and death.[11–13] These reports, however, have largely relied on analysis of administrative level data from linked electronic records, and lacked clinical granularity to explore patients' in-hospital features and their outcomes.

Using comprehensive clinical patient level data from consecutive patients admissions to Imperial College Healthcare NHS Trust (ICHNT), one of the largest hospital Trusts in England serving a multi-ethnic population of over 600,000 in North West London, [14] we aimed to: 1) describe baseline characteristics and outcomes for patients hospitalized with laboratory-confirmed SARS-CoV-2 infection early in the pandemic; 2) evaluate the proportion of patients hospitalized at ICHNT for COVID-19 who are from BAME groups and whether ethnicity is associated with mortality; and 3) evaluate clinical and laboratory observations on admission associated with mortality. With a wealth of clinical trials underway, baseline analyses such as this will serve as a useful comparison for the results of interventional studies.

## 2. Methods

### Study Setting

We performed a retrospective cohort study at ICHNT, including all admissions with polymerase chain reaction (RT-PCR)-positive SARS-CoV-2 infection between February 25$^{th}$ and April 5$^{th}$, 2020. The cohort opened at the time of admission and censoring was done as of May 1$^{st}$, 2020. We excluded those who did not initially present with symptoms suggestive of COVID-19, those with clinical suspicion of COVID-19 but negative RT-PCR, those who did not require hospitalization and transfers from other hospitals. Where a patient had two or more RT-PCR positive results, we included the first episode that required hospitalization.

### Ethics

The study was approved by the ICHNT clinical governance team. As we report on routinely collected non-identifiable clinical audit data, no ethical approval was required under the UK policy framework for health and social care.

### Data Collection

Individual patient electronic medical records were used to extract demographic, clinical, laboratory, radiological, comorbidity and outcome data including need for ICU admission and invasive ventilation, using a standardized data template. Demographic characteristics included patients' sex, age, ethnicity and whether they were a health worker. Pre-existing comorbidities were recorded individually and used to calculate the Elixhauser comorbidity score, a points-based system validated in the UK to evaluate risk of hospital mortality.[15,16] Ethnicity is normally registered on initial presentation to the Emergency department for all patients at ICHNT. Given challenges with capacity and infection control at ICHNT during the present epidemic, this variable was often recorded as 'other-ethnicity'. For completeness, these entries were checked against London Ambulance Service records, previous clinic letters and GP referral letters and reclassified as appropriate. Where ethnicity was still unable to be confirmed, we report this as unknown.

We recorded clinical observations at the time of initial presentation to hospital, including New Early Warning Score (NEWS-2), respiratory rate, mean arterial blood pressure, temperature, oxygen saturations (SaO2) and oxygen requirements. SARS-CoV-2 testing was performed by RT-PCR of specimens collected by nasopharyngeal swabs. Laboratory results of serum creatinine, urea, glucose and lactate were only included if performed within 4 hours of admission. All other blood tests and chest radiography were included if performed within 24 hours of admission. Chest radiography findings were reported as per recommendations from the British Society of Thoracic Imaging Guidelines (see Supplement).[17]

**Outcomes of interest and statistical analysis**

We assessed the primary outcome of vital status as discharged alive or died and an intermediate outcome of clinical deterioration, defined as either requiring a 60% or greater inspiratory fraction of oxygen to maintain SaO2 > 94%, invasive ventilation or admission to

ICU. We used standard chi squared, t-student or Wilcoxon rank sum tests, as appropriate, to describe the prevalence of covariates at admission among patients with a completed outcome.

We further performed adjusted and unadjusted logistic regression to assess a) predictors of COVID-19 hospital mortality and b) differences in mortality by ethnicity. Numeric variables (e.g. age, hemoglobin, creatinine, etc.) were treated as continuous if their density distribution plot showed a normal distribution in either the natural or logarithmic scale, else they were coded into categorical. Variables with a *p*-value below 0.157 in unadjusted regression were selected for adjusted regression, as per the Akaike Information Criterion.[18] In the adjusted regression model, variables were considered statistically significant with a *p*-value <0.05 and thus kept as final candidates in the prediction model. Variables with more than 20% missing values were excluded from regression analysis.

We evaluated time from hospital admission to clinical deterioration and final outcome accounting for competing risks and right-censored data (i.e. patients still in hospital at the time of censoring) using the Nelson-Aelen and Kaplan-Meier estimators, respectively (Supplement). The effect of selected covariates on the hazard of clinical deterioration and death was assessed using Cox Proportional-Hazard models. The proportional hazards assumptions were evaluated using Schoenfeld's residuals.[19]

All analyses were performed using R Studio (version 1.2.5033). The funders of this study had no role in the study design, data collection, analysis, interpretation, or reporting.

**3. Results**

**Description of cohort**

756 patients had a positive SARS-CoV-2 nasopharyngeal swab and were hospitalized at ICHNT between 25th February 2020 and 5th April 2020, of which 142 were excluded as their clinical presentation was for other non-COVID-19 symptoms (but subsequently tested positive for SARS-CoV-2 during hospitalization). Of 614 patients included in analyses, 559 had completed outcomes and 55 (9%) were still hospitalized at the date of censoring (Figure 1). Their median age was 69 years (IQR 25), 382 (62%) were male, 235 (38%) were white, 244 (40%) were from a BAME group and for 135 (22%) the ethnic background was unknown.

The mean time from symptom onset to hospital admission was 7.2 days (SD 5.6), with cough, fever and shortness of breath being highly prevalent at presentation, and 192 (31%) had gastrointestinal symptoms. On initial clinical assessment, 196 patients (32%) had an NEWS-2 score ≥7, with 420 (69%) having a SaO2 <95% on room air or requiring supplemental oxygen to maintain normal SaO2. Amongst patients with full blood count available ($n$=605), 262 (43%) had anemia <130g/L, 385 (64%) had lymphopenia <1.1 x $10^9$/L, and 97 (16%) had thrombocytopenia <130 x $10^9$/L. Biochemical and inflammatory marker abnormalities were also found in a high proportion of patients, most notably a reduced glomerular filtration rate (eGFR) and a raised C-reactive protein (CRP) (Table 1). Abnormalities in their inflammatory and cardiac injury markers (e.g. D-dimer, troponin) were also highly prevalent, albeit these investigations were significantly under-recorded and thus not included in regression analyses (Table 1).

Nearly half the cohort ($n$=276) received at least 60% oxygen during their admission and 80 (13%) received invasive ventilation. Of patients with completed outcomes, 381 (68%) were discharged alive and 178 (32%) patients died in hospital. The median length of hospital stay, accounting for patients with pending outcomes, was 7 days (IQR 6-8) and the median time to clinical deterioration was 15 days (IQR 8-32) (Supplementary Figure S2).

***Predictors of mortality***

Out of those with completed outcomes (*n*=559 patients), the median age, sex and comorbidity profiles were significantly different between those who died and those who were discharged alive (Table 1 and Supplementary Table S1). In unadjusted logistic regression, male sex (OR 1.69, 95% CI 1.15-2.47) and age (OR 1.05, 95% CI 1.04-1.06) were strongly associated with increased odds death (Table 2 and Supplementary Figure S3). Other admission characteristics significantly associated with increased mortality were having an NEWS-2 ≥7 (OR 3.79, 95% CI 2.40-6.00), body temperature >38˚C (OR 1.83, 95% CI 1.25-2.67) and having high oxygen requirements (i.e. either SaO2 <95% despite supplementary oxygen < 60% [OR 2.77, 95% CI 1.42-5.39)] or any SaO2 measurement whilst on FiO2 ≥ 60% [OR 5.41, 95% CI 3.17-9.25]).

Also in unadjusted regression, thrombocytopenia (increase OR 1.00, 95% CI 1.00-1.01, per x$10^9$L decrease), lymphopenia <0.5 x $10^9$/L (OR 2.67, 95% CI 1.41-5.210), leukocytosis ≥10.6 x $10^9$/L (OR 1.99, 95% CI 1.24-3.19), eGFR <30mL/min/1.73$m^2$ (OR 8.57, 95% CI 4.43-16.57), low albumin (increase OR 1.09, 95% CI 1.04-1.14, per g/dL decrease) and C-reactive protein ≥100mg/dL (OR 7.22, 95%CI 2.16-24.13) were associated with increased probability of death.

Hypertension, diabetes, ischemic heart disease, atrial fibrillation, chronic kidney disease and dementia had an unadjusted association with mortality (Table 2). However, when accounting for age, only diabetes (aOR 1.47, 95%CI 1.00-2.16) and CKD (aOR 1.86, 95%CI 1.15-3.00)

remained statistically significant. Asthma appeared to be a protective factor for death both in unadjusted regression and after adjusting for age (aOR 0.42, 95%CI 0.19-0.91) (Table 2).

In adjusted logistic regression, clinical and laboratory observations with an increased odds ratio of mortality that remained statistically significant were fever (≥38˚C) on admission, having a $SaO_2$ <95% whilst on supplementary oxygen or if more that 60% $FiO_2$ was administered on admission, elevated white cell count, thrombocytopenia (continuous), hypoalbuminemia (continuous) and a reduced eGFR (Table 2) (area under the receiver operating curve [AUROC] 0.77) (Table S5).

***<u>Outcomes by ethnicity</u>***

235 (38%) patients were of white background, 133 (22%) black, 94 (15%) Asian, 17 (3%) 'other' and 135 (22%) of unknown ethnicity. Overall, neither the intermediate (requirement of $FiO_2$ ≥60% or invasive ventilation) nor final outcomes (death or discharged alive) were significantly different between ethnic groups (Table 3). However, white patients were significantly older, with an average age of 70.6 years (compared to 63.6 years for black, 66.4 Asian and 54.8 for 'other' ethnicity) (p<0.01) and also had a higher burden of pre-existing comorbidities as defined by the Elixhauser score ($p$=0.02) (Table 3 and Supplementary Figure S4).

Despite their younger age composition and lower Elixhauser score, the severity of disease on presentation (NEWS-2) was similar between patients of BAME and white ethnicity ($p$=0.28) (Table 3 and Supplementary Figure S4). Furthermore, when stratifying ethnic groups by the median age of the overall cohort (i.e. 69 years), we found that the cumulative probability of death of black patients was in fact higher than of their white counterparts (Figure 2).

We thus performed unadjusted and adjusted logistic regression analyses to assess the odds of death of BAME groups compared to white patients. The unadjusted odds of death did not differ across ethnic groups (Table 3 and Supplementary Table S4). However, when adjusting for age, sex, comorbidities and NEWS-2 on admission, the odds of death of black compared to white patients was higher (aOR 1.85 95% CI 1.06-3.24, p=0.04, AUROC 0.78) (Table 3). If admission severity by NEWS-2 was removed from the regression model, the trend remained albeit less strongly (aOR 1.69, 95%CI 1.00-2.86, p=0.049, AUROC 0.73) (Supplementary Table S4). Clinically relevant, albeit marginally non-statistically significant, trends were observed for Asian (aOR 1.78, 95%CI 0.97-3.29, p=0.06) patients and those of unknown ethnicity (Table 3). The proportion of patients with clinical and laboratory abnormalities on admission associated with increased odds of death also varied by ethnic group (Supplementary Figure S6). Notably eGFR alterations ($p$<0.01) and fever ($p$=0.01) were more prevalent amongst BAME groups. The prevalence of the rest of the prognostic markers did not show significant variations by ethnicity.

**4. Discussion**

We present detailed demographic, clinical and outcome data for 614 laboratory-confirmed SARS-CoV-2 cases at a large multi-ethnic London NHS Trust. Our study provides methodologically robust evidence that, beyond the widely reported factors associated with increased odds of COVID-19 mortality (age, sex and severe hypoxemia on admission), thrombocytopenia, leukocytosis, hypoalbuminemia and reduced eGFR are also significantly associated with increased in-hospital death. Furthermore, we present an in-depth analysis of the clinical association between ethnicity and patient outcomes during the COVID-19

epidemic in the UK, a current topic of high global health priority. In addition to an over-representation of BAME patients in our hospitalized COVID-19 cohort (when compared to historic admissions to ICHNT – Supplementary Table S3), we find an association of increased odds of death amongst black (compared to white) patients, when adjusted for age, sex, burden of comorbidities and severity of disease on admission .

Emerging evidence on the associations between ethnicity and COVID-19 outcomes has been conflicting. In the UK, reports from grey literature highlight a higher risk of death for BAME than for white patients, which has been partially explained by sociodemographic differences and pre-existing co-morbidities.[8,11] A ONS report, however, relied on historical population census data (self-reported health indices) to determine these associations,[11] and a pre-print study focused on comorbidities without admission severity or other clinical observations.[8] Our study thus adds an important clinical perspective, using comprehensive granular patient level data, which national datasets cannot often provide.

We find that the underlying profiles of the different ethnic groups varied, with black and 'other' ethnic minority groups being younger, and Asian and white groups having a higher burden of pre-existing comorbidities. A high prevalence of undiagnosed comorbidities amongst BAME groups has been previously identified,[20] albeit the significance of this in the current pandemic is yet unknown. We observed that after adjusting for potential confounders, such as age, sex, comorbidities and severity of admission, black (compared to white) patients were at increased odds of death. This clinically relevant trend was also seen for Asian patients, although marginally non-statistically significant. Interestingly, another recent UK study found ethnicity to be one of eight covariates predicting ICU admission but not mortality.[21] In that study, though, the majority of non-white patients were black, whereas our study had high representation of both black and Asian patients. Together, our findings and those from other emerging studies suggest important biological factors may be potentially driving differences in COVID-19 hospitalization outcomes by ethnicity, which cannot solely be explained by socioeconomic differences.

The symptom profile in our cohort was largely consistent with previous studies.[4–6,12,21] However, both in our study and others from the UK,[12] a much higher proportion of patients reported shortness of breath on admission compared with what has been reported in China (65-67*% vs* 25%).[22] Furthermore, a higher proportion of our cohort showed abnormal inflammatory markers and thrombocytopenia. These discrepancies could reflect differing thresholds and criteria for hospitalization between the UK and other countries. High mortality rates have been seen in many settings, for example 26% amongst all admissions in an Italian study and 88% for mecanically ventilated patients in another from the USA.[6,23] We found a crude mortality rate of 29% overall and of 52% amongst ventilated patients, albeit 55 patients in our study remained hospitalized at the time of censoring. The extent to whether inter-country variations in clinical practice and capacity restraints affects in-hospital mortality is currently unknown and warrants further investigation.

A key strength of our study is that we have systematically identified associations between demographic and clinical factors associated with outcomes in a cohort of over 600 patients through multiple regression, survival and competing risks analyses. Previous studies have focused on descriptive statistics,[4–6,12,22] with few robustly correcting for covariates in their evaluation of outcomes.[21]

However, limitations to our study should be acknowledged. First, although we attempted to be as comprehensive as possible, the retrospective nature of this study limited the availability of data. Missing values, including of key variables such as BMI, ethnicity and pre-existing comorbidities, could be possibly related to the severity of disease on admission and we may have thus missed important associations with mortality. We tried to overcome these data limitations as thoroughly as possible by cross-checking medical clerking on admission and discharge, with patients' referral letters and historic records, where available. Also, many of the non-routine laboratory tests (including D-dimer, BNP and troponin) were only introduced systematically at ICHNT a few weeks into the pandemic. Although descriptive statistics suggest there are differences in the prevalence of these variables by patient outcomes, they were excluded from the multiple logistic regression given incomplete recording. Strengthening of data recording has been implemented in our hospitals. This, together with standardized laboratory investigation protocols, will improve future observational studies, as well as the monitorization of patient outcomes in intervention trials.

Finally, this study did not consider the characteristics and outcomes of those hospitalized with clinical features of COVID-19 but were RT-PCR swab-negative and those who might have had hospital-acquired SARS-CoV-2 infection. The profiles of these patients and whether their outcomes are different to the patients presented here requires further research. Furthermore, we could not quantify the impact of changes in clinical practice over the time of our study. However, only a few patients were enrolled in clinical trials early in the pandemic so changing clinical practices are unlikely to impact on our study findings.

**Conclusions**

This is one of the first studies in the UK to analyze the associations between demographic and clinical characteristics of COVID-19 patients and their hospitalization outcomes by ethnicity. We have employed robust methodological analyses to account for the effect of patients with uncompleted outcomes. The admission characteristics with the strongest association with increased odds of death were fever, severe hypoxemia, thrombocytopenia, leukocytosis, hypoalbuminemia and a reduced eGFR. BAME groups were overrepresented in our study population, compared with historical admissions to our hospital trust. After accounting for age, comorbidity profile and severity of disease on admission, patients of black and Asian ethnicity appear to have worst hospitalization outcomes. Research is urgently needed into complex multifactorial interactions underpinning differences in COVID-19 disease outcomes by ethnicity. Such analyses would have a vital role in informing changes in clinical and public health practice locally and abroad.

## Notes

### Author contributions

SN, KH, TBH, PP conceived the study.

SN and PG wrote the first manuscript draft.

PP, MK and MV analyzed the data.

PA and PW provided methodological expertise.

SN, PP, MRT, PA, PW, KH, TBH interpreted the data.

SN, PP, AD, SM, RF, PC, PM, RJ, CH, AS collected the data.

SN, PP, MRT and GC provided clinical expertise.

All authors reviewed and edited the manuscript for scientific content.

### Acknowledgements

We would like to acknowledge J-IDEA, Imperial BRC Centre and MRC GIDA for providing the platforms for multi-collaborative projects such as this one. The authors would like to thank all patients at Imperial College Healthcare NHS Trust and its staff, who have been involved in the management of patients during this pandemic, in particular, Julian Redhead, Tim Orchard, Georgie Russell, Claire Ross, Susie Bloch, Ernie Wong, Simon Ashworth and Carlos Gomez. We would also like to thank Jonathan Clarke, Luca Mercuri, Ben Glampson and Dimitri Papadimiriou for providing data support for the analysis.

### Disclaimer

The study sponsors had no role in study design, data collection, data analysis, data interpretation, report writing, or decision to submit for publication. The views expressed are those of the authors and not necessarily those of the funders. The corresponding author had full access to all study data and had final responsibility for the decision to submit for publication.

## Financial support

We acknowledge funding from the MRC Centre for Global Infectious Disease Analysis (MR/R015600/1); this award is jointly funded by the UK Medical Research Council (MRC) and the UK Department for International Development (DFID) under the MRC/DFID Concordat agreement and is also part of the EDCTP2 program supported by the European Union (EU). KH acknowledges joint Centre funding from the Abdul Latif Jameel Institute for Disease and Emergency Analytics (J-IDEA).

**Conflicts of interest**: P.A. reports a research grant from Dr Foster Ltd (A wholly owned subsidiary of Telstra Health). None of the other authors have any potential conflicts.

**Figure legends**

**Figure 1: Overview of cohort and patient pathways.**

Last included patients as of the 5th of April and last recorded outcomes as of the 1st of May 2020

**Figure 2**: **Cumulative probability of hospitalization outcome amongst those older than the median age of the cohort (i.e. 69 years) by ethnic groups.**

Calculated with Nelson-Aeler estimator. Note from text and Table 3 that white patients were significantly older than BAME groups.

*p<0.05

*Abbreviations: BAME, black, Asian and other ethnic minority groups.*

## Table legends

**Table 1**: Clinical characteristics of COVID-19 patients at ICHNT

*Variables excluded from regression analysis due to greater than 20% missing values

**Analyzed with Kaplan-Meier estimator. Values for *All* account for censoring of those with uncompleted outcomes. Values for *Died* and *Survived* restricted to those with completed outcomes. P values as assessed with Cox proportional hazards models and Schoenfeld's residuals. For *Survived*, estimation of time to clinical deterioration was not possible with these methods, as less than 50% in the subgroup had the event of interest.

***Chest radiograph classification as per the British Society of Thoracic Imaging, whereby 0 = normal, 1 = classic COVID-19 findings, 2= abnormal findings indeterminate for COVID-19, and 3 = non-COVID-19 findings

*Abbreviations: ALP, alkaline phosphatase; BMI, body mass index; BP, blood pressure; CK, creatine kinase; CRP, C-reactive protein; eGFR, estimated glomerular filtration rate; FiO2, inspiratory fraction of oxygen; ICU, intensive care unit; IU, international units; IQR, interquartile range; LDH, lactate dehydrogenase; MAP, mean arterial pressure; MDRD, modification of diet in renal disease; NEWS-2, New Early Warning Score; PT, prothrombin time; SD, standard deviation; WCC, white cell count*

**Table 2**: Logistic regression for clinical and laboratory predictors of hospital death

*p<0.05, **p<0.01, ***p<0.001

†Adjusted regression model for clinical and laboratory observations on admission. Only variables that remained statistically significant are shown. For full model selection process, see Tables S5 in Supplementary material. Final model's AUROC = 0.77

Δ NEWS-2 was not included in adjusted model, as this is already a measure of patient severity based on clinical observations (i.e. consciousness, respiratory rate, SaO2, pulse, systolic blood pressure, supplementary oxygen and temperature).

*Abbreviations: 95%CI, 95% confidence interval; AUROC, area under the receiver operating curve; BMI, body mass index; BNP, brain natriuretic peptide; BP, blood pressure; CK, creatine kinase; CKD, chronic kidney disease; COPD, chronic obstructive pulmonary disease; CRP, C-reactive protein; DVT/PE, deep vein thrombosis / pulmonary embolism; NEWS-2, New Early Warning Score; eGFR, estimated glomerular filtration rate; FiO2, inspiratory fraction of oxygen; IU, international units; LDH, lactate dehydrogenase; MDRD, modification of diet in renal disease; OR, odds ratio; SaO2, oxygen saturation; WCC, white cell count*

**Table 3**: Clinical characteristics by ethnicity and logistic regression of odds of death

*p<0.05

†Adjusted regression model for age, male sex, Elixhauser score and NEWS-2 differences by ethnic groups. Additional logistic regression models adjusted for any comorbidity, diabetes and CKD were also carried out, but were inferior predictors compared to the selected model. COPD, cirrhotic liver disease and HIV/AIDS were not used in logistic regression models, as they had low *n* values for ethnic groups

*Abbreviations: CKD, chronic kidney disease; COPD, chronic obstructive pulmonary disease; DVT/PE, deep vein thrombosis / pulmonary embolism; NEWS-2, New Early Warning Score; ICU, intensive care unit; HIV/AIDS, human immunodeficiency virus / acquired immunodeficiency syndrome; IVS, invasive ventilation support; PVD, peripheral vascular disease; SD, standard deviation*

**Table 1**: Clinical characteristics of COVID-19 patients at ICHNT

*Variables excluded from regression analysis due to greater than 20% missing values

**Analyzed with Kaplan-Meier estimator. Values for *All* account for censoring of those with uncompleted outcomes. Values for *Died* and *Survived* restricted to those with completed outcomes. P values as assessed with Cox proportional hazards models and Schoenfeld's residuals. For *Survived*, estimation of time to clinical deterioration was not possible with these methods, as less than 50% in the subgroup had the event of interest.

***Chest radiograph classification as per the British Society of Thoracic Imaging, whereby 0 = normal, 1 = classic COVID-19 findings, 2= abnormal findings indeterminate for COVID-19, and 3 = non-COVID-19 findings

*Abbreviations: ALP, alkaline phosphatase; BMI, body mass index; BP, blood pressure; CK, creatine kinase; CRP, C-reactive protein; eGFR, estimated glomerular filtration rate; FiO2, inspiratory fraction of oxygen; ICU, intensive care unit; IU, international units; IQR, interquartile range; LDH, lactate dehydrogenase; MAP, mean arterial pressure; MDRD, modification of diet in renal disease; NEWS-2, New Early Warning Score; PT, prothrombin time; SD, standard deviation; WCC, white cell count*

| | | **All (n = 614)** | **Died (n = 178)** | **Survived (n = 381)** | **p value** |
|---|---|---|---|---|---|
| **Demography** | | | | | |
| Male, n (%) | | 382 (62.21%) | 125 (70.22%) | 222 (58.27%) | <0.01 |
| Median age (IQR) | | 69 (25) | 77 (17) | 63 (28) | <0.01 |
| Mean BMI (SD)* | | 28.85 (7.23) | 29.47 (8.23) | 28.62 (6.76) | 0.38 |
| Median Elixhauser (IQR) | | 2.5 (9.75) | 6 (11) | 0 (7) | <0.01 |
| Ethnicity | White (%) | 235 (38.27%) | 67 (37.64%) | 149 (39.11%) | 0.81 |
| | Black (%) | 133 (21.66%) | 41 (23.03%) | 78 (20.47%) | 0.56 |
| | Asian (%) | 94 (15.31%) | 30 (16.85%) | 54 (14.17%) | 0.48 |

| | | | | | |
|---|---|---|---|---|---|
| | Other (%) | 17 (2.77%) | 3 (1.69%) | 14 (3.67%) | 0.31 |
| | Missing (%) | 135 (21.99%) | 37 (20.79%) | 86 (22.57%) | 0.71 |
| Healthcare worker, n (%) | | 23 (3.75%) | 3 (1.69%) | 17 (4.46%) | 0.16 |
| **Symptoms** | | | | | |
| Mean (SD) days prior to admission | | 7.2 (5.6) | 6.0 (5.4) | 7.7 (5.6) | <0.01 |
| Cough, n (%) | | 453 (73.78%) | 120 (67.42%) | 287 (75.33%) | 0.06 |
| Fever, n (%) | | 501 (81.60%) | 147 (82.58%) | 310 (81.36%) | 0.82 |
| Shortness of breath, n (%) | | 400 (65.15%) | 128 (71.91%) | 235 (61.68%) | 0.02 |
| Gastrointestinal, n (%) | | 192 (31.27%) | 43 (24.16%) | 136 (35.70%) | <0.01 |
| Other, n (%) | | 401 (65.31%) | 101 (56.74%) | 264 (69.29%) | <0.01 |
| **Outcomes** | | | | | |
| Received >60% FiO2 (%) | | 276 (44.95%) | 142 (79.78%) | 96 (25.20%) | <0.01 |
| Admitted to ICU (%) | | 87 (14.17%) | 28 (15.73%) | 24 (6.30%) | <0.01 |
| Received invasive ventilation (%) | | 80 (13.03%) | 24 (13.48%) | 22 (5.77%) | <0.01 |
| Median length of hospital stay (IQR)** | | 7 (6-8) | 7 (5-8) | 6 (5-7) | 1.00 |
| Median time to clinical deterioration (IQR)** | | 15 (8-32) | 2 (1-4) | NA | <0.01 |
| **Clinical observations on admissions** | | | | | |
| Fever >=38°C | | 162/607 (26.69%) | 60/177 (33.90%) | 85/378 (22.49%) | <0.01 |
| Respiratory rate | <20 | 167/598 (27.93%) | 42/175 (24.00%) | 115/371 (31.00%) | 0.11 |

| | | | | | |
|---|---|---|---|---|---|
| | 20 - 29 | 308/598 (51.51%) | 86/175 (49.14%) | 197/371 (53.10%) | 1.00 |
| | ≥30 | 123/598 (20.57%) | 47/175 (26.86%) | 59/371 (15.90%) | <0.01 |
| SaO2 | ≥95% on room air | 189/609 (31.03%) | 31 (17.42%) | 143/378 (37.83%) | <0.01 |
| | < 95% and/or on supplemental oxygen | 420/609 (68.97%) | 147 (82.58%) | 235/378 (62.17%) | <0.01 |
| MAP | ≥100 | 207/606 (34.16%) | 56/177 (31.64%) | 126/376 (33.51%) | 0.73 |
| | 70 - 99 | 363/606 (59.90%) | 105/177 (59.32%) | 232/376 (61.70%) | 0.66 |
| | < 90 | 36/606 (5.94%) | 16/177 (9.04%) | 18/376 (4.79%) | 0.08 |
| Pulse | ≥100 | 234/609 (38.42%) | 67/177 (37.85%) | 141/379 (37.20%) | 0.96 |
| | 60 -99 | 367/609 (60.26%) | 108/177 (61.02%) | 232/379 (61.21%) | 1.00 |
| | < 60 | 8/609 (1.31%) | 2/177 (1.13%) | 6/379 (1.58%) | 0.97 |
| NEWS-2 | ≥7 | 196/610 (32.13%) | 87 (48.88%) | 86/379 (22.69%) | <0.01 |
| | 5 - 6 | 137/610 (22.46%) | 35 (19.66%) | 94/379 (24.80%) | 0.22 |
| | <5 | 277/610 (45.41%) | 56 (31.46%) | 199/379 (52.51%) | <0.01 |
| **Bloods** | | | | | |
| Hemoglobin g/L | ≥130 | 343/605 (56.69%) | 88/173 (50.87%) | 219/378 (57.94%) | 0.14 |
| | 100 - 129 | 225/605 (37.19%) | 69/173 (39.88%) | 141/378 (37.30%) | 0.63 |
| | <100 | 37/605 (6.12%) | 16/173 (9.25%) | 18/378 (4.76%) | 0.07 |

| | | | | | |
|---|---|---|---|---|---|
| White cell count x 109L | ≥10.6 | 101/605 (16.69%) | 39/173 (22.54%) | 48/378 (12.70%) | <0.01 |
| | 4.2 - 10.5 | 449/605 (74.21%) | 120/173 (69.36%) | 292/378 (77.25%) | 0.05 |
| | <4.2 | 55/605 (9.09%) | 15/173 (8.67%) | 39/378 (10.32%) | 0.65 |
| Lymphocytes x 109L | ≥ 3·6 | 11/604 (1.82%) | 2/173 (1.16%) | 6/377 (1.59%) | 0.99 |
| | 1·1 - 3·5 | 208/604 (34.44%) | 46/173 (26.59%) | 144/377 (38.20%) | 0.01 |
| | 0·5 - 1·0 | 334/604 (55.30%) | 102/173 (58.96%) | 200/377 (53.05%) | 0.23 |
| | < 0·5 | 51/604 (8.44%) | 23/173 (13.29%) | 27/377 (7.16%) | 0.03 |
| Platelets x 109L | ≥ 370 | 32/603 (5.31%) | 7/171 (4.09%) | 21/378 (5.56%) | 0.61 |
| | 130 - 369 | 486/603 (80.60%) | 122/171 (71.35%) | 311/378 (82.28%) | <0.01 |
| | < 130 | 97/603 (16.09%) | 42/171 (24.56%) | 46/378 (12.17%) | <0.01 |
| Creatinine mmol/L | ≥ 125 | 160/601 (26.62%) | 70/172 (40.70%) | 73/375 (19.47%) | <0.01 |
| | < 125 | 441/601 (73.38%) | 102/172 (59.30%) | 302/375 (80.53%) | <0.01 |
| Urea mmol/L | ≥ 7·8 | 222/598 (37.12%) | 93/171 (54.39%) | 111/374 (29.68%) | <0.01 |
| | < 7·8 | 376/598 (62.88%) | 78/171 (45.61%) | 263/374 (70.32%) | <0.01 |
| eGFR MDRD ml/min/1.73m2 | ≥ 90 | 155/587 (26.41%) | 17/168 (10.12%) | 124/365 (33.97%) | <0.01 |
| | 60 - 89 | 201/587 (34.24%) | 57/168 (33.93%) | 128/365 (35.07%) | 1.00 |
| | 30 - 59 | 137/587 (23.34%) | 47/168 (27.98%) | 73/365 (20.00%) | 0.04 |
| | < 30 | 94/587 (16.01%) | 47/168 (27.98%) | 40/365 (10.96%) | <0.01 |

| | | | | | |
|---|---|---|---|---|---|
| Albumin g/L | ≥ 35 | 135/539 (25.05%) | 28/149 (18.79%) | 102/339 (30.09%) | 0.01 |
| | 25 - 34 | 347/539 (64.38%) | 102/149 (68.46%) | 207/339 (61.06%) | 0.01 |
| | < 25 | 57/539 (10.58%) | 19/149 (12.75%) | 30/339 (8.85%) | 0.25 |
| ALT IU/L | ≥ 3x ULN | 17/535 (3.18%) | 5/150 (3.33%) | 11/333 (3.30%) | 1.00 |
| | 1 - 2·9x ULN | 129/535 (24.11%) | 28/150 (18.67%) | 82/333 (24.62%) | 0.18 |
| | < 1x ULN | 389/535 (72.71%) | 117/150 (78.00%) | 240/333 (72.07%) | 0.21 |
| Bilirubin mmol/L | ≥ 21 | 64/520 (12.31%) | 23/143 (16.08%) | 32/326 (9.82%) | 0.07 |
| | < 21 | 456/520 (87.69%) | 120/143 (83.92%) | 294/326 (90.18%) | 0.07 |
| ALP IU/L | ≥ 130 | 77/549 (14.03%) | 34/154 (22.08%) | 35/343 (10.20%) | <0.01 |
| | < 130 | 472/549 (85.97%) | 120/154 (77.92%) | 308/343 (89.80%) | <0.01 |
| PT seconds | ≥ 17·4 | 45/452 (9.96%) | 18/125 (14.40%) | 24/280 (8.57%) | 0.11 |
| | < 17·4 | 407/452 (90.04%) | 107/125 (85.60%) | 256/280 (91.43%) | 0.11 |
| Lactate* mmol/L | ≥ 2 | 119/490 (24.29%) | 47/143 (32.87%) | 59/298 (19.80%) | <0.01 |
| | < 2 | 371/490 (75.71%) | 96/143 (67.13%) | 239/298 (80.20%) | <0.01 |
| Glucose mmol/L | ≥ 5·2 | 463/507 (91.32%) | 136/149 (91.28%) | 281/308 (91.23%) | 1.00 |
| | 3·7 - 5·1 | 226/507 (44.58%) | 12/149 (8.05%) | 28/308 (9.09%) | 0.84 |
| | < 3·7 | 5/507 (0.99%) | 2/149 (1.34%) | 1/308 (0.32%) | 0.52 |
| CRP mg/L | ≥ 100 | 309/589 (52.46%) | 106/167 (63.47%) | 163/370 (44.05%) | <0.01 |
| | 10 -99 | 243/589 (41.26%) | 58/167 (34.73%) | 174/370 (47.03%) | 0.01 |
| | < 10 | 37/589 (6.28%) | 3/167 (1.80%) | 33/370 (8.92%) | <0.01 |

| | | | | | |
|---|---|---|---|---|---|
| D-dimer* ng/mL | ≥ 3000 | 56/304 (18.42%) | 20/89 (22.47%) | 29/180 (16.11%) | 0.27 |
| | 2000 - 2999 | 36/304 (11.84%) | 16/89 (17.98%) | 17/180 (9.44%) | 0.07 |
| | 1000 - 1999 | 90/304 (29.61%) | 23/89 (25.84%) | 52/180 (28.89%) | 0.70 |
| | 500 - 999 | 85/304 (27.96%) | 23/89 (25.84%) | 54/180 (30.00%) | 0.57 |
| | < 500 | 37/304 (12.17%) | 7/89 (7.87%) | 28/180 (15.56%) | 0.12 |
| LDH* IU/L | ≥ 243 | 224/244 (91.80%) | 64/69 (92.75%) | 133/146 (91.10%) | 0.88 |
| | < 243 | 20/244 (8.20%) | 5/69 (7.25%) | 13/146 (8.90%) | 0.88 |
| Troponin* ng/L | ≥ 34 | 124/406 (30.54%) | 61/119 (51.26%) | 48/243 (19.75%) | <0.01 |
| | < 34 | 282/406 (69.46%) | 58/119 (48.74%) | 196/243 (80.66%) | <0.01 |
| CK* U/L | ≥ 320 | 90/294 (30.61%) | 37/86 (43.02%) | 42/175 (24.00%) | <0.01 |
| | < 320 | 204/294 (69.39%) | 49/86 (56.98%) | 133/175 (76.00%) | <0.01 |
| BNP* pg/mL | ≥ 150 | 48/261 (18.39%) | 23/75 (30.67%) | 21/162 (12.96%) | <0.01 |
| | < 150 | 213/261 (81.61%) | 52/75 (69.33%) | 141/162 (87.04%) | <0.01 |
| Ferritin* ng/mL | >=5000 | 14/350 (4.00%) | 4/103 (3.88%) | 9/206 (4.37%) | 1.00 |
| | 1000 - 4999 | 130/350 (37.14%) | 38/103 (36.89%) | 70/206 (33.98%) | 0.70 |
| | 500 - 999 | 96/350 (27.43%) | 30/103 (29.13%) | 55/206 (26.70%) | 0.75 |
| | 300 - 499 | 50/350 (14.29%) | 13/103 (12.62%) | 32/206 (15.53%) | 0.61 |
| | < 300 | 60/350 (17.14%) | 18/103 (17.48%) | 40/206 (19.42%) | 0.80 |
| Cortisol* nmol/L | ≥ 550 | 127/188 (67.55%) | 42/57 (73.68%) | 70/111 (63.06%) | 0.23 |
| | < 550 | 61/188 (32.45%) | 15/57 (26.32%) | 41/111 (36.94%) | 0.23 |

| Chest X-ray | | | | | |
|---|---|---|---|---|---|
| 0 | | 76/499 (15.23%) | 15/148 (10.14%) | 59/310 (19.03%) | 0.02 |
| 1 | Mild | 28/610 (4.59%) | 5 (2.81%) | 20/377 (5.31%) | 0.27 |
| | Moderate | 115/605 (19.01%) | 33/175 (18.86%) | 74/376 (19.68%) | 0.91 |
| | Severe | 104/607 (17.13%) | 31/174 (17.82%) | 53/380 (13.95%) | 0.29 |
| 2 | Mild | 48/610 (7.87%) | 12 (6.74%) | 34/377 (9.02%) | 0.46 |
| | Moderate | 69/605 (11.40%) | 28/175 (16.00%) | 35/376 (9.31%) | 0.03 |
| | Severe | 12/607 (1.98%) | 7/174 (4.02%) | 5/380 (1.32%) | 0.09 |
| 3 | | 26/499 (5.21%) | 12/148 (8.11%) | 14/310 (4.52%) | 0.18 |

*p<0.05, **p<0.01, ***p<0.001

†Adjusted regression model for clinical and laboratory observations on admission. Only variables that remained statistically significant are shown. For full model selection process, see Tables S5 in Supplementary material. Final model's AUROC = 0.77

Δ NEWS-2 was not included in adjusted model, as this is already a measure of patient severity based on clinical observations (i.e. consciousness, respiratory rate, SaO2, pulse, systolic blood pressure, supplementary oxygen and temperature).

*Abbreviations: 95%CI, 95% confidence interval; AUROC, area under the receiver operating curve; BMI, body mass index; BNP, brain natriuretic peptide; BP, blood pressure; CK, creatine kinase; CKD, chronic kidney disease; COPD, chronic obstructive pulmonary disease; CRP, C-reactive protein; DVT/PE, deep vein thrombosis / pulmonary embolism; NEWS-2, New Early Warning Score; eGFR, estimated glomerular filtration rate; FiO2, inspiratory fraction of oxygen; IU, international units; LDH, lactate dehydrogenase; MDRD, modification of diet in renal disease; OR, odds ratio; SaO2, oxygen saturation; WCC, white cell count*

| | **Unadjusted** | **Adjusted for age** |
|---|---|---|
| **Demography** | | |
| Age | **1.05 *** [1.04, 1.06]** | - |
| Male sex | **1.69 ** [1.15, 2.47]** | **1.90 ***[1.27, 2.85]** |
| Asian vs white | 1.24 [0.73, 2.10] | 1.59 [0.90, 2.81] |
| Black vs white | 1.17 [0.73, 1.88] | 1.58 [0.95, 2.64] |
| Missing vs white | 0.96 [0.59, 1.55] | 1.50 [0.89, 2.53] |
| Other mixed vs white | 0.48 [0.13, 1.71] | 1.02 [0.27, 3.90] |
| **Pre-existing chronic diseases** | | |
| Any comorbidity | **3.60 *** [2.08, 6.23]** | 1.81 [1.00, 3.29] |

| | | |
|---|---|---|
| Hypertension | **1.89 *** [1.32, 2.72]** | 1.26 [0.86, 1.86] |
| Diabetes | **1.68 ** [1.16, 2.42]** | **1.47* [1.00, 2.16]** |
| Ischemic heart disease | **2.29 ** [1.39, 3.76]** | 1.45 [0.86, 2.44] |
| Chronic heart failure | 1.99 [0.99, 4.00] | 1.25 [0.61, 2.59] |
| Stroke | 1.33 [0.74, 2.40] | 0.89 [0.48, 1.65] |
| Chronic kidney disease | **2.55 *** [1.62, 4.01]** | **1.86* [1.15, 3.00]** |
| Dementia | **2.67 *** [1.58, 4.50]** | 1.32 [0.75, 2.32] |
| DVT/PE (previous) | 2.9 [0.64, 13.08] | 3.07 [0.65, 14.40] |
| Atrial fibrillation | **1.68 * [1.01, 2.80]** | 1.25 [0.73, 2.13] |
| COPD | 1.07 [0.47, 2.44] | 0.78 [0.34, 1.81] |
| Asthma | **0.44 * [0.21, 0.93]** | **0.42* [0.19, 0.91]** |
| Liver disease (non-cirrhotic) | 0.77 [0.38, 1.58] | 0.99 [0.47, 2.11] |
| Liver disease (cirrhotic) | 3.08 [0.96, 9.84] | 3.19 [0.95, 10.76] |
| Solid malignant tumor | 1.21 [0.68, 2.16] | 0.74 [0.40, 1.35] |
| Hematologic malignancy | 1.29 [0.30, 5.45] | 1.24 [0.28, 5.44] |
| HIV/AIDS | 0.85 [0.16, 4.45] | 1.32 [0.24, 7.36] |
| | **Unadjusted** | **Adjusted † model** |
| **Clinical observations on admission** | | |
| Fever | **1.83 ** [1.25, 2.67]** | **1.82 ** [1.18, 2.80]** |
| Respiratory <20 (intercept) | | |

| | | | |
|---|---|---|---|
| rate | | | |
| | 20-29 | 1.2 [0.77, 1.85] | - |
| | ≥30 | **2.18 ** [1.30, 3.67]** | - |
| SaO2 | ≥95% on room air (intercept) | | |
| | <95% on room air | **2.14 * [1.17, 3.93]** | 1.82 [0.96, 3.47] |
| | ≥95% on FiO2 < 60% | **1.96 * [1.14, 3.35]** | **1.88 * [1.05, 3.37]** |
| | <95% on FiO2 < 60% | **2.77 ** [1.42, 5.39]** | **2.67 ** [1.31, 5.45]** |
| | Any on FiO2 ≥ 60% | **5.41 *** [3.17, 9.25]** | **4.25 *** [2.36, 7.64]** |
| MAP | 70-99 (intercept) | | |
| | <70 | 1.96 [0.96, 4.00] | - |
| | ≥100 | 0.98 [0.66, 1.45] | - |
| Pulse | 60-99 (intercept) | | |
| | <60 | 0.72 [0.14, 3.61] | - |
| | ≥100 | 1.02 [0.71, 1.48] | - |
| NEWS-2 Δ | <4 (intercept) | | |
| | 4-6 | 1.34 [0.83, 2.15] | - |
| | ≥7 | **3.79 *** [2.40, 6.00]** | - |
| **Laboratory investigations on admission** | | | |
| Hemoglobin | ≥130 (intercept) | | |
| | 100-129 | **2.21 * [1.08, 4.53]** | - |

| | | | |
|---|---|---|---|
| | <100 | 1.22 [0.83, 1.78] | - |
| White cells | 4.2-10.5 (intercept) | | |
| | <4.2 | 0.94 [0.50, 1.77] | 0.78 [0.38, 1.59] |
| | ≥10.6 | **1.99 ** [1.24, 3.19]** | **2.35 ** [1.35, 4.11]** |
| Lymphocytes | 1.1-3.4 (intercept) | | |
| | <0.5 | **2.67 ** [1.40, 5.10]** | - |
| | 0.5-1.0 | **1.60 * [1.06, 2.40]** | - |
| | ≥3.5 | 1.04 [0.20, 5.35] | - |
| Platelets (numeric) | | **1.00 ** [1.00,1.01]** | **1.01 *** [1.00, 1.01]** |
| Albumin (numeric) | | **1.09 *** [1.04,1.12]** | **1.06 *** [1.02, 1.09]** |
| Bilirubin (numeric) | | 1.01 [0.99, 1.03] | - |
| ALP (numeric) | | 1.00 [1.00, 1.00] | - |
| Creatinine | <125 (intercept) | | |
| | ≥125 | **2.84 *** [1.91, 4.22]** | - |
| eGFR | ≥90 (intercept) | | |
| | 60-89 | **3.25 *** [1.79, 5.89]** | **2.65 ** [1.47, 4.78]** |
| | 30-59 | **4.70 *** [2.51, 8.78]** | **3.78 *** [2.03, 7.04]** |
| | <30 | **8.57 *** [4.43, 16.57]** | **5.14 *** [2.65, 9.97]** |
| Glucose | 3.7-5.1 (intercept) | | |

| | | | |
|---|---|---|---|
| | <3.7 | 4.73 [0.39, 57.70] | - |
| | ≥5.2 | 1.14 [0.55, 2.38] | - |
| | Missing | 0.94 [0.41, 2.14] | - |
| CRP | <10 (intercept) | | |
| | 10-99 | **3.67 * [1.08, 12.40]** | - |
| | ≥100 | **7.15 ** [2.14, 23.90]** | - |
| | Missing | **11.00 ** [2.59, 46.76]** | - |
| CXR | Normal or non-COVID findings (intercept) | | |
| | Mild | 0.85 [0.42, 1.72] | - |
| | Moderate/severe | 1.54 [0.93, 2.54] | - |
| | Missing | 1.14 [0.62, 2.11] | - |

*p<0.05

†Adjusted regression model for age, male sex, Elixhauser score and NEWS-2 differences by ethnic groups. Additional logistic regression models adjusted for any comorbidity, diabetes and CKD were also carried out, but were inferior predictors compared to the selected model. COPD, cirrhotic liver disease and HIV/AIDS were not used in logistic regression models, as they had low *n* values for ethnic groups

*Abbreviations: CKD, chronic kidney disease; COPD, chronic obstructive pulmonary disease; DVT/PE, deep vein thrombosis / pulmonary embolism; NEWS-2, New Early Warning Score; ICU, intensive care unit; HIV/AIDS, human immunodeficiency virus / acquired immunodeficiency syndrome; IVS, invasive ventilation support; PVD, peripheral vascular disease; SD, standard deviation*

| | **White (n = 235)** | **Black (n = 133)** | **Asian (n = 94)** | **Other (n = 17)** | **Missing (n = 135)** | **p value** |
|---|---|---|---|---|---|---|
| Male, n (%) | 146 (62.13%) | 76 (57.14%) | 60 (63.83%) | 12 (70.59%) | 88 (65.19%) | 0.63 |
| Mean age (SD) | 70.52 (15.69) | 63.58 (19.08) | 66.4 (15.88) | 54.82 (15.25) | 60.7 (18.11) | <0.01 |
| Mean Elixhauser score (SD) | 6.29 (7.25) | 5.23 (6.85) | 6.63 (7.15) | 2.53 (4.37) | 2.99 (5.12) | <0.01 |
| Mean NEWS-2 score (SD) | 5.09 (3.2) | 4.93 (3.04) | 4.74 (3.31) | 5.41 (2.67) | 5.39 (2.83) | 0.28 |
| Mean days to admission (SD) | 6.99 (5.75) | 7.33 (5.98) | 6.15 (4.82) | 7.29 (6.47) | 8.16 (5.08) | 0.49 |
| **Outcomes** | | | | | | |
| FiO2 >=60%, n (%) | 100 (42.55%) | 61 (45.86%) | 43 (45.74%) | 5 (29.41%) | 70 (51.85%) | 0.31 |
| Admitted ICU, n (%) | 23 (9.79%) | 20 (15.04%) | 15 (15.96%) | 2 (11.76%) | 27 (20.00%) | 0.10 |
| Received IVS, n (%) | 22 (9.36%) | 18 (13.53%) | 14 (14.89%) | 2 (11.76%) | 24 (17.78%) | 0.21 |
| Died in hospital, n (%) | 67 (28.51%) | 41 (30.83%) | 30 (31.91%) | 3 (17.65%) | 37 (27.41%) | 0.76 |
| Discharged alive, n (%) | 149 (63.40%) | 78 (58.65%) | 54 (57.45%) | 14 (82.35%) | 86 (63.70%) | 0.31 |

| | | | | | | |
|---|---|---|---|---|---|---|
| Pending outcome, n (%) | 19 (8.09%) | 14 (10.53%) | 10 (10.64%) | 0 (0.00%) | 12 (8.89%) | 0.62 |
| **Comorbidities** | | | | | | |
| Any comorbidity, n (%) | 179 (76.17%) | 112 (84.21%) | 78 (82.98%) | 11 (64.71%) | 100 (74.07%) | 0.10 |
| Ischemic heart disease, n (%) | 33 (14.04%) | 16 (12.03%) | 18 (19.15%) | 0 (0.00%) | 13 (9.63%) | 0.12 |
| Chronic heart failure, n (%) | 19 (8.09%) | 11 (8.27%) | 1 (1.06%) | 0 (0.00%) | 6 (4.44%) | 0.07 |
| Hypertension, n (%) | 97 (41.28%) | 73 (54.89%) | 40 (42.55%) | 7 (41.18%) | 69 (51.11%) | 0.08 |
| Hyperlipidemia, n (%) | 57 (24.26%) | 32 (24.06%) | 27 (28.72%) | 2 (11.76%) | 33 (24.44%) | 0.66 |
| Diabetes, n (%) | 61 (25.96%) | 63 (47.37%) | 41 (43.62%) | 4 (23.53%) | 47 (34.81%) | <0.01 |
| Chronic kidney disease, n (%) | 39 (16.60%) | 26 (19.55%) | 26 (27.66%) | 0 (0.00%) | 11 (8.15%) | <0.01 |
| Peripheral vascular disease, n (%) | 11 (4.68%) | 3 (2.26%) | 0 (0.00%) | 0 (0.00%) | 4 (2.96%) | 0.19 |
| Stroke, n (%) | 28 (11.91%) | 13 (9.77%) | 11 (11.70%) | 1 (5.88%) | 4 (2.96%) | 0.05 |
| Atrial fibrillation, n (%) | 43 (18.30%) | 10 (7.52%) | 11 (11.70%) | 2 (11.76%) | 9 (6.67%) | <0.01 |
| DVT/PE history, n (%) | 4 (1.70%) | 2 (1.50%) | 0 (0.00%) | 0 (0.00%) | 1 (0.74%) | 0.68 |
| Hemiplegia, n (%) | 6 (2.55%) | 0 (0.00%) | 0 (0.00%) | 0 (0.00%) | 0 (0.00%) | 0.04 |
| Dementia, n (%) | 42 (17.87%) | 9 (6.77%) | 10 (10.64%) | 0 (0.00%) | 9 (6.67%) | <0.01 |
| Asthma, n (%) | 18 (7.66%) | 14 (10.53%) | 8 (8.51%) | 3 (17.65%) | 13 (9.63%) | 0.65 |
| COPD, n (%) | 22 (9.36%) | 3 (2.26%) | 3 (3.19%) | 0 (0.00%) | 2 (1.48%) | <0.01 |
| Connective tissue disease, n (%) | 4 (1.70%) | 3 (2.26%) | 1 (1.06%) | 0 (0.00%) | 1 (0.74%) | 0.82 |
| Peptic ulcer, n (%) | 8 (3.40%) | 2 (1.50%) | 1 (1.06%) | 0 (0.00%) | 3 (2.22%) | 0.61 |

| | | | | | | |
|---|---|---|---|---|---|---|
| Liver (non-cirrhotic), n (%) | 14 (5.96%) | 12 (9.02%) | 10 (10.64%) | 3 (17.65%) | 5 (3.70%) | 0.09 |
| Liver (cirrhotic), n (%) | 2 (0.85%) | 4 (3.01%) | 6 (6.38%) | 0 (0.00%) | 1 (0.74%) | 0.02 |
| Solid tumor, n (%) | 33 (14.04%) | 9 (6.77%) | 8 (8.51%) | 1 (5.88%) | 8 (5.93%) | 0.06 |
| Hematologic tumor, n (%) | 4 (1.70%) | 1 (0.75%) | 1 (1.06%) | 0 (0.00%) | 2 (1.48%) | 0.92 |
| HIV/AIDS, n (%) | 1 (0.43%) | 7 (5.26%) | 1 (1.06%) | 0 (0.00%) | 0 (0.00%) | <0.01 |
| **Logistic regression of odds of death by ethnicity** | | | | | | |
| Unadjusted OR (95%CI) | Intercept | 1.17 [0.73, 1.88] | 1.24 [0.73, 2.10] | 0.48 [0.13, 1.71] | 0.96 [0.59, 1.55] | |
| Adjusted† model OR (95%CI) | Intercept | 1.85 * [1.06, 3.24] | 1.78 [0.97, 3.29] | 0.95 [0.24, 3.80] | 1.63 [0.93, 2.84] | |

Figure 1

Accepted

Figure 2

Accepted
