# Clinical Characteristics and Predictors of Outcomes of Hospitalized Patients With Coronavirus Disease 2019 in a Multiethnic London National Health Service Trust: A Retrospective Cohort Study

**DOI:** 10.1093/cid/ciaa1091

**Published:** 2020-08-07

**Authors:** Pablo N Perez-Guzman, Anna Daunt, Sujit Mukherjee, Peter Crook, Roberta Forlano, Mara D Kont, Alessandra Løchen, Michaela Vollmer, Paul Middleton, Rebekah Judge, Christopher Harlow, Anet Soubieres, Graham Cooke, Peter J White, Timothy B Hallett, Paul Aylin, Neil Ferguson, Katharina Hauck, Mark R Thursz, Shevanthi Nayagam

**Affiliations:** 1 Medical Research Council Centre for Global Infectious Disease Analysis and Abdul Latif Jameel Institute for Disease and Emergency Analytics, School of Public Health, Imperial College London, London, United Kingdom; 2 Division of Digestive Diseases, Department of Metabolism, Digestion and Reproduction, Faculty of Medicine, Imperial College London, London, United Kingdom; 3 Imperial College Healthcare National Health Service Trust, London, United Kingdom; 4 Department of Infectious Diseases, Imperial College London, London, United Kingdom; 5 Department of Primary Care and Public Health, School of Public Health, Imperial College London, London, United Kingdom

**Keywords:** COVID-19, mortality, ethnic minority groups

## Abstract

**Background:**

Emerging evidence suggests ethnic minorities are disproportionately affected by coronavirus disease 2019 (COVID-19). Detailed clinical analyses of multicultural hospitalized patient cohorts remain largely undescribed.

**Methods:**

We performed regression, survival, and cumulative competing risk analyses to evaluate factors associated with mortality in patients admitted for COVID-19 in 3 large London hospitals between 25 February and 5 April, censored as of 1 May 2020.

**Results:**

Of 614 patients (median age, 69 [interquartile range, 25] years) and 62% male), 381 (62%) were discharged alive, 178 (29%) died, and 55 (9%) remained hospitalized at censoring. Severe hypoxemia (adjusted odds ratio [aOR], 4.25 [95% confidence interval {CI}, 2.36–7.64]), leukocytosis (aOR, 2.35 [95% CI, 1.35–4.11]), thrombocytopenia (aOR [1.01, 95% CI, 1.00–1.01], increase per 10^9^ decrease), severe renal impairment (aOR, 5.14 [95% CI, 2.65–9.97]), and low albumin (aOR, 1.06 [95% CI, 1.02–1.09], increase per gram decrease) were associated with death. Forty percent (n = 244) were from black, Asian, and other minority ethnic (BAME) groups, 38% (n = 235) were white, and ethnicity was unknown for 22% (n = 135). BAME patients were younger and had fewer comorbidities. Although the unadjusted odds of death did not differ by ethnicity, when adjusting for age, sex, and comorbidities, black patients were at higher odds of death compared to whites (aOR, 1.69 [95% CI, 1.00–2.86]). This association was stronger when further adjusting for admission severity (aOR, 1.85 [95% CI, 1.06–3.24]).

**Conclusions:**

BAME patients were overrepresented in our cohort; when accounting for demographic and clinical profile of admission, black patients were at increased odds of death. Further research is needed into biologic drivers of differences in COVID-19 outcomes by ethnicity.

## Teaser

Fever, hypoxemia, thrombocytopenia, hypoalbuminemia, leukocytosis, and renal impairment on admission are associated with increased odds of hospital mortality among COVID-19 patients. Black ethnic background is an independent predictor of hospital mortality, after adjusting for demographic and clinical confounders.

The United Kingdom (UK) has been the third worst affected country in the world by coronavirus disease 2019 (COVID-19), with a reported death toll of 46 413 as of 6 August 2020 [1]. Most deaths have occurred in London [2], a densely populated, multicultural capital city where >40% of residents identify themselves as belonging to a black, Asian, or other ethnic minority (BAME) [3].

Previous studies have found that age, sex, and comorbidities including cardiovascular disease are associated with poorer COVID-19 hospitalization outcomes [4–8]. However, robust analyses adjusting for confounding factors and evaluating the association of the physiological and laboratory characteristics on admission with clinical outcomes are lacking. Such studies are urgently needed to allow frontline clinicians to tailor their management decisions and hospital policymakers to efficiently plan their response to COVID-19.

Data from UK admission to intensive care units (ICUs) and from the Office for National Statistics suggest that BAME groups are more frequently affected with COVID-19 than white patients [9, 10]. This is largely thought to be due to economic deprivation driving an increased risk of COVID-19 infection and death [11–13]. These reports, however, have largely relied on analysis of administrative-level data from linked electronic records, and lack clinical granularity to explore patients’ in-hospital features and their outcomes.

Using comprehensive clinical patient-level data from consecutive patient admissions to the Imperial College Healthcare National Health Service Trust (ICHNT), one of the largest hospital Trusts in England serving a multiethnic population of >600 000 in North West London [14], we aimed to (1) describe baseline characteristics and outcomes for patients hospitalized with laboratory-confirmed severe acute respiratory syndrome coronavirus 2 (SARS-CoV-2) infection early in the pandemic; (2) evaluate the proportion of patients hospitalized at ICHNT for COVID-19 who are from BAME groups and whether ethnicity is associated with mortality; and (3) evaluate clinical and laboratory observations on admission associated with mortality. With a wealth of clinical trials underway, baseline analyses such as this will serve as a useful comparison for the results of interventional studies.

## METHODS

### Study Setting

We performed a retrospective cohort study at ICHNT, including all admissions with reverse-transcription polymerase chain reaction (RT-PCR)–positive SARS-CoV-2 infection between 25 February and 5 April 2020. The cohort opened at the time of admission and censoring was done as of 1 May 2020. We excluded those who did not initially present with symptoms suggestive of COVID-19, those with clinical suspicion of COVID-19 but negative RT-PCR, those who did not require hospitalization, and transfers from other hospitals. Where a patient had 2 or more RT-PCR positive results, we included the first episode that required hospitalization.

### Ethical Approvals

The study was approved by the ICHNT clinical governance team. As we report on routinely collected nonidentifiable clinical audit data, no ethical approval was required under the UK policy framework for health and social care.

### Data Collection

Individual patient electronic medical records were used to extract demographic, clinical, laboratory, radiological, comorbidity, and outcome data including need for ICU admission and invasive ventilation, using a standardized data template. Demographic characteristics included patients’ sex, age, ethnicity, and whether they were a health worker. Pre-existing comorbidities were recorded individually and used to calculate the Elixhauser comorbidity score, a points-based system validated in the UK to evaluate risk of hospital mortality [15, 16]. Ethnicity is normally registered on initial presentation to the emergency department for all patients at ICHNT. Given challenges with capacity and infection control at ICHNT during the present epidemic, this variable was often recorded as “other ethnicity.” For completeness, these entries were checked against London Ambulance Service records, previous clinic letters and GP referral letters, and reclassified as appropriate. Where ethnicity was still unable to be confirmed, we report this as unknown.

We recorded clinical observations at the time of initial presentation to hospital, including New Early Warning Score (NEWS-2), respiratory rate, mean arterial blood pressure, temperature, oxygen saturation (SaO_2_), and oxygen requirements. SARS-CoV-2 testing was performed by RT-PCR of specimens collected by nasopharyngeal swabs. Laboratory results of serum creatinine, urea, glucose, and lactate were only included if performed within 4 hours of admission. All other blood tests and chest radiography were included if performed within 24 hours of admission. Chest radiographic findings were reported as per recommendations from the British Society of Thoracic Imaging guidelines (Supplementary Data) [17].

### Outcomes of Interest and Statistical Analysis

We assessed the primary outcome of vital status as discharged alive or died and an intermediate outcome of clinical deterioration, defined as either requiring a ≥60% fraction of inspired oxygen (FiO_2_) to maintain SaO_2_ >94%, invasive ventilation, or admission to ICU. We used standard χ ^2^, Student *t*, or Wilcoxon rank-sum tests, as appropriate, to describe the prevalence of covariates at admission among patients with a completed outcome.

We further performed adjusted and unadjusted logistic regression to assess (1) predictors of COVID-19 hospital mortality and (2) differences in mortality by ethnicity. Numeric variables (eg, age, hemoglobin, creatinine) were treated as continuous if their density distribution plot showed a normal distribution in either the natural or logarithmic scale, else they were coded into categorical. Variables with a *P* value below .157 in unadjusted regression were selected for adjusted regression, as per the Akaike information criterion [18]. In the adjusted regression model, variables were considered statistically significant with a *P* value <.05 and thus kept as final candidates in the prediction model. Variables with >20% missing values were excluded from regression analysis.

We evaluated time from hospital admission to clinical deterioration and final outcome accounting for competing risks and right-censored data (ie, patients still in hospital at the time of censoring) using the Nelson-Aalen and Kaplan-Meier estimators, respectively (Supplementary Data). The effect of selected covariates on the hazard of clinical deterioration and death was assessed using Cox proportional hazard models. The proportional hazards assumptions were evaluated using Schoenfeld residuals [19].

All analyses were performed using R Studio (version 1.2.5033). The funders of this study had no role in the study design, data collection, analysis, interpretation, or reporting.

## RESULTS

### Description of Cohort

**Figure 1.**
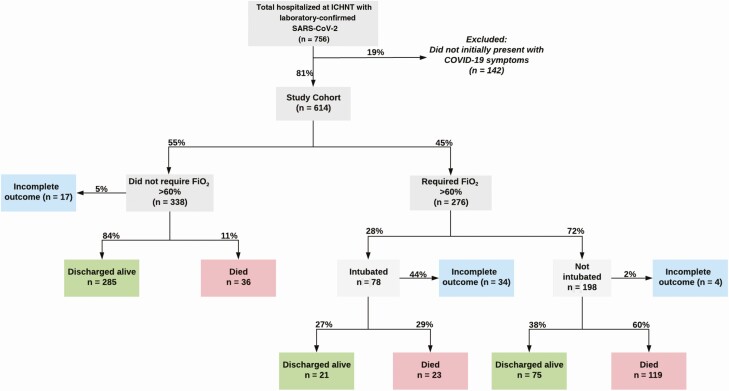
Overview of cohort and patient pathways. Last included patients as of 5 April and last recorded outcomes as of 1 May 2020. Abbreviations: COVID-19, coronavirus disease 2019; FiO_2_, fraction of inspired oxygen; ICHNT, Imperial College Healthcare National Health Service Trust; SARS-CoV-2, severe acute respiratory syndrome coronavirus 2.

Seven hundred fifty-six patients had a positive SARS-CoV-2 nasopharyngeal swab and were hospitalized at ICHNT between 25 February and 5 April 2020, of which 142 were excluded as their clinical presentation was for other non-COVID-19 symptoms (but subsequently tested positive for SARS-CoV-2 during hospitalization). Of 614 patients included in analyses, 559 had completed outcomes and 55 (9%) were still hospitalized at the date of censoring (Figure 1). Their median age was 69 years (interquartile range [IQR], 25), 382 (62%) were male, 235 (38%) were white, 244 (40%) were from a BAME group, and for 135 (22%) the ethnic background was unknown.

The mean time from symptom onset to hospital admission was 7.2 days (standard deviation, 5.6 days), with cough, fever, and shortness of breath being highly prevalent at presentation; 192 (31%) had gastrointestinal symptoms. On initial clinical assessment, 196 patients (32%) had NEWS-2 of ≥7, with 420 (69%) having an SaO_2_ <95% on room air or requiring supplemental oxygen to maintain normal SaO_2_. Among patients with full blood count available (n = 605), 262 (43%) had anemia <130 g/L, 385 (64%) had lymphopenia <1.1 × 10^9^/L, and 97 (16%) had thrombocytopenia <130 × 10^9^/L. Biochemical and inflammatory marker abnormalities were also found in a high proportion of patients, most notably a reduced glomerular filtration rate (eGFR) and a raised C-reactive protein (CRP) (Table 1). Abnormalities in their inflammatory and cardiac injury markers (eg, D-dimer, troponin) were also highly prevalent, albeit these investigations were significantly underrecorded and thus not included in regression analyses (Table 1).

**Table 1.** Clinical Characteristics of Patients With Coronavirus Disease 2019 at Imperial College Healthcare National Health Service Trust

Characteristic	All (N = 614)	Died (n = 178)	Survived (n = 381)	*P* Value
Demography				
Male sex	382 (62.21)	125 (70.22)	222 (58.27)	<.01
Median age, y (IQR)	69 (54–79)	77 (67–84)	63 (50–78)	<.01
Mean BMI, kg/m^2^ (SD)^a^	28.85 (7.23)	29.47 (8.23)	28.62 (6.76)	.38
Median Elixhauser score (IQR)	2.5 (0–9.75)	6 (0–11)	0 (0–7)	<.01
Ethnicity				
White	235 (38.27)	67 (37.64)	149 (39.11)	.81
Black	133 (21.66)	41 (23.03)	78 (20.47)	.56
Asian	94 (15.31)	30 (16.85)	54 (14.17)	.48
Other	17 (2.77)	3 (1.69)	14 (3.67)	.31
Missing	135 (21.99)	37 (20.79)	86 (22.57)	.71
Healthcare worker	23 (3.75)	3 (1.69)	17 (4.46)	.16
Symptoms				
Mean (SD) days prior to admission	7.2 (5.6)	6.0 (5.4)	7.7 (5.6)	<.01
Cough	453 (73.78)	120 (67.42)	287 (75.33)	.06
Fever	501 (81.60)	147 (82.58)	310 (81.36)	.82
Shortness of breath	400 (65.15)	128 (71.91)	235 (61.68)	.02
Gastrointestinal	192 (31.27)	43 (24.16)	136 (35.70)	<.01
Other	401 (65.31)	101 (56.74)	264 (69.29)	<.01
Outcomes				
Received >60% FiO_2_	276 (44.95)	142 (79.78)	96 (25.20)	<.01
Admitted to ICU	87 (14.17)	28 (15.73)	24 (6.30)	<.01
Received invasive ventilation	80 (13.03)	24 (13.48)	22 (5.77)	<.01
Median length of hospital stay (IQR)^d^	7 (6–8)	7 (5–8)	6 (5–7)	1.00
Median time to clinical deterioration (IQR)^d^	15 (8–32)	2 (1–4)	NA	<.01
Clinical observations on admission				
Fever ≥38°C	162/607 (26.69)	60/177 (33.90)	85/378 (22.49)	<.01
Respiratory rate, breaths/min				
<20	167/598 (27.93)	42/175 (24.00)	115/371 (31.00)	.11
20–29	308/598 (51.51)	86/175 (49.14)	197/371 (53.10)	1.00
≥30	123/598 (20.57)	47/175 (26.86)	59/371 (15.90)	<.01
SaO_2_				
≥95% on room air	189/609 (31.03)	31 (17.42)	143/378 (37.83)	<.01
<95% and/or on supplemental oxygen	420/609 (68.97)	147 (82.58)	235/378 (62.17)	<.01
MAP, mm Hg				
≥100	207/606 (34.16)	56/177 (31.64)	126/376 (33.51)	.73
70–99	363/606 (59.90)	105/177 (59.32)	232/376 (61.70)	.66
<90	36/606 (5.94)	16/177 (9.04)	18/376 (4.79)	.08
Pulse, beats/min				
≥100	234/609 (38.42)	67/177 (37.85)	141/379 (37.20)	.96
60–99	367/609 (60.26)	108/177 (61.02)	232/379 (61.21)	1.00
<60	8/609 (1.31)	2/177 (1.13)	6/379 (1.58)	.97
NEWS-2				
≥7	196/610 (32.13)	87 (48.88)	86/379 (22.69)	<.01
5–6	137/610 (22.46)	35 (19.66)	94/379 (24.80)	.22
<5	277/610 (45.41)	56 (31.46)	199/379 (52.51)	<.01
Blood tests				
Hemoglobin, g/L				
≥130	343/605 (56.69)	88/173 (50.87)	219/378 (57.94)	.14
100–129	225/605 (37.19)	69/173 (39.88)	141/378 (37.30)	.63
<100	37/605 (6.12)	16/173 (9.25)	18/378 (4.76)	.07
White cell count, ×10^9^ cells/L				
≥10.6	101/605 (16.69)	39/173 (22.54)	48/378 (12.70)	<.01
4.2–10.5	449/605 (74.21)	120/173 (69.36)	292/378 (77.25)	.05
<4.2	55/605 (9.09)	15/173 (8.67)	39/378 (10.32)	.65
Lymphocytes, ×10^9^ cells/L				
≥3.6	11/604 (1.82)	2/173 (1.16)	6/377 (1.59)	.99
1.1–3.5	208/604 (34.44)	46/173 (26.59)	144/377 (38.20)	.01
0.5–1.0	334/604 (55.30)	102/173 (58.96)	200/377 (53.05)	.23
<0.5	51/604 (8.44)	23/173 (13.29)	27/377 (7.16)	.03
Platelets, × 10^9^/L				
≥370	32/603 (5.31)	7/171 (4.09)	21/378 (5.56)	.61
130–369	486/603 (80.60)	122/171 (71.35)	311/378 (82.28)	<.01
<130	97/603 (16.09)	42/171 (24.56)	46/378 (12.17)	<.01
Creatinine, mmol/L				
≥125	160/601 (26.62)	70/172 (40.70)	73/375 (19.47)	<.01
<125	441/601 (73.38)	102/172 (59.30)	302/375 (80.53)	<.01
Urea, mmol/L				
≥7.8	222/598 (37.12)	93/171 (54.39)	111/374 (29.68)	<.01
<7.8	376/598 (62.88)	78/171 (45.61)	263/374 (70.32)	<.01
eGFR (MDRD), mL/min/1.73 m^2^				
≥90	155/587 (26.41)	17/168 (10.12)	124/365 (33.97)	<.01
60–89	201/587 (34.24)	57/168 (33.93)	128/365 (35.07)	1.00
30–59	137/587 (23.34)	47/168 (27.98)	73/365 (20.00)	.04
<30	94/587 (16.01)	47/168 (27.98)	40/365 (10.96)	<.01
Albumin, g/L				
≥35	135/539 (25.05)	28/149 (18.79)	102/339 (30.09)	.01
25–34	347/539 (64.38)	102/149 (68.46)	207/339 (61.06)	.01
<25	57/539 (10.58)	19/149 (12.75)	30/339 (8.85)	.25
ALT, IU/L				
≥3 × ULN	17/535 (3.18)	5/150 (3.33)	11/333 (3.30)	1.00
1–2.9 × ULN	129/535 (24.11)	28/150 (18.67)	82/333 (24.62)	.18
<1 × ULN	389/535 (72.71)	117/150 (78.00)	240/333 (72.07)	.21
Bilirubin mmol/L				
≥21	64/520 (12.31)	23/143 (16.08)	32/326 (9.82)	.07
<21	456/520 (87.69)	120/143 (83.92)	294/326 (90.18)	.07
ALP, IU/L				
≥130	77/549 (14.03)	34/154 (22.08)	35/343 (10.20)	<.01
<130	472/549 (85.97)	120/154 (77.92)	308/343 (89.80)	<.01
Prothrombin time, sec				
≥17.4	45/452 (9.96)	18/125 (14.40)	24/280 (8.57)	.11
<17.4	407/452 (90.04)	107/125 (85.60)	256/280 (91.43)	.11
Lactate^a^, mmol/L				
≥2	119/490 (24.29)	47/143 (32.87)	59/298 (19.80)	<.01
<2	371/490 (75.71)	96/143 (67.13)	239/298 (80.20)	<.01
Glucose, mmol/L				
≥5.2	463/507 (91.32)	136/149 (91.28)	281/308 (91.23)	1.00
3.7–5.1	226/507 (44.58)	12/149 (8.05)	28/308 (9.09)	.84
<3.7	5/507 (0.99)	2/149 (1.34)	1/308 (0.32)	.52
CRP, mg/L				
≥100	309/589 (52.46)	106/167 (63.47)	163/370 (44.05)	<.01
10–99	243/589 (41.26)	58/167 (34.73)	174/370 (47.03)	.01
<10	37/589 (6.28)	3/167 (1.80)	33/370 (8.92)	<.01
D-dimer^a^, ng/mL				
≥3000	56/304 (18.42)	20/89 (22.47)	29/180 (16.11)	.27
2000–2999	36/304 (11.84)	16/89 (17.98)	17/180 (9.44)	.07
1000–1999	90/304 (29.61)	23/89 (25.84)	52/180 (28.89)	.70
500–999	85/304 (27.96)	23/89 (25.84)	54/180 (30.00)	.57
<500	37/304 (12.17)	7/89 (7.87)	28/180 (15.56)	.12
LDH^a^, IU/L				
≥243	224/244 (91.80)	64/69 (92.75)	133/146 (91.10)	.88
<243	20/244 (8.20)	5/69 (7.25)	13/146 (8.90)	.88
Troponin^a^, ng/L				
≥34	124/406 (30.54)	61/119 (51.26)	48/243 (19.75)	<.01
<34	282/406 (69.46)	58/119 (48.74)	196/243 (80.66)	<.01
Creatine kinase^a^, U/L				
≥320	90/294 (30.61)	37/86 (43.02)	42/175 (24.00)	<.01
<320	204/294 (69.39)	49/86 (56.98)	133/175 (76.00)	<.01
BNP^a^, pg/mL				
≥150	48/261 (18.39)	23/75 (30.67)	21/162 (12.96)	<.01
<150	213/261 (81.61)	52/75 (69.33)	141/162 (87.04)	<.01
Ferritin^a^, ng/mL				
≥5000	14/350 (4.00)	4/103 (3.88)	9/206 (4.37)	1.00
1000–4999	130/350 (37.14)	38/103 (36.89)	70/206 (33.98)	.70
500–999	96/350 (27.43)	30/103 (29.13)	55/206 (26.70)	.75
300–499	50/350 (14.29)	13/103 (12.62)	32/206 (15.53)	.61
<300	60/350 (17.14)	18/103 (17.48)	40/206 (19.42)	.80
Cortisol^a^, nmol/L				
≥550	127/188 (67.55)	42/57 (73.68)	70/111 (63.06)	.23
<550	61/188 (32.45)	15/57 (26.32)	41/111 (36.94)	.23
Chest radiograph^c^				
0 (normal)	76/499 (15.23)	15/148 (10.14)	59/310 (19.03)	.02
1 (classic COVID-19 findings)				
Mild	28/610 (4.59)	5 (2.81)	20/377 (5.31)	.27
Moderate	115/605 (19.01)	33/175 (18.86)	74/376 (19.68)	.91
Severe	104/607 (17.13)	31/174 (17.82)	53/380 (13.95)	.29
2 (abnormal; indeterminate for COVID-19)				
Mild	48/610 (7.87)	12 (6.74)	34/377 (9.02)	.46
Moderate	69/605 (11.40)	28/175 (16.00)	35/376 (9.31)	.03
Severe	12/607 (1.98)	7/174 (4.02)	5/380 (1.32)	.09
3 (non–COVID-19 findings)	26/499 (5.21)	12/148 (8.11)	14/310 (4.52)	.18

Data are presented as no. (%) unless otherwise indicated.

Abbreviations: ALP, alkaline phosphatase; ALT, alanine aminotransferase; BMI, body mass index; BNP, brain natriuretic peptide; COVID-19 coronavirus disease 2019; CRP, C-reactive protein; eGFR, estimated glomerular filtration rate; FiO_2_, inspiratory fraction of oxygen; ICU, intensive care unit; IU, international units; IQR, interquartile range; LDH, lactate dehydrogenase; MAP, mean arterial pressure; MDRD, Modification of Diet in Renal Disease; NA, not available; NEWS-2, New Early Warning Score; SD, standard deviation; ULN, upper limit of normal.

^a^Variables excluded from regression analysis due to >20% missing values.

^b^Analyzed with Kaplan-Meier estimator. Values for “All” account for censoring of those with uncompleted outcomes. Values for “Died” and “Survived” restricted to those with completed outcomes. *P* values as assessed with Cox proportional hazards models and Schoenfeld residuals. For “Survived,” estimation of time to clinical deterioration was not possible with these methods, as <50% in the subgroup had the event of interest.

^c^Chest radiograph classification as per the British Society of Thoracic Imaging.

Nearly half of the cohort (n* *= 276) received at least 60% oxygen during their admission and 80 (13%) received invasive ventilation. Of patients with completed outcomes, 381 (68%) were discharged alive and 178 (32%) patients died in hospital. The median length of hospital stay, accounting for patients with pending outcomes, was 7 days (IQR, 6–8 days) and the median time to clinical deterioration was 15 days (IQR, 8–32 days) (Supplementary Figure 2).

### Predictors of Mortality

Out of those with completed outcomes (n = 559 patients), the median age, sex, and comorbidity profiles were significantly different between those who died and those who were discharged alive (Table 1 and Supplementary Table 1). In unadjusted logistic regression, male sex (odds ratio [OR], 1.69 [95% confidence interval {CI}, 1.15–2.47]) and age (OR, 1.05 [95% CI, 1.04–1.06]) were strongly associated with increased odds of death (Table 2 and Supplementary Figure 3). Other admission characteristics significantly associated with increased mortality were NEWS-2 ≥7 (OR, 3.79 [95% CI, 2.40–6.00]), body temperature >38°C (OR, 1.83 [95% CI, 1.25–2.67]), and having high oxygen requirements (ie, either SaO_2_ <95% despite supplementary oxygen <60%; OR, 2.77 [95% CI, 1.42–5.39]) or any SaO_2_ measurement while on FiO_2_ ≥60% (OR, 5.41 [95% CI, 3.17–9.25]).

**Table 2.** Logistic Regression for Clinical and Laboratory Predictors of Hospital Death

Characteristic	Unadjusted	Adjusted for Age
Demography		
Age	1.05*** (1.04–1.06)	…
Male sex	1.69** (1.15–2.47)	1.90***(1.27–2.85)
Asian vs white	1.24 (.73–2.10)	1.59 (.90–2.81)
Black vs white	1.17 (.73–1.88)	1.58 (.95–2.64)
Missing vs white	0.96 (.59–1.55)	1.50 (.89–2.53)
Other mixed vs white	0.48 (.13–1.71)	1.02 (.27–3.90)
Pre-existing chronic diseases		
Any comorbidity	3.60*** (2.08–6.23)	1.81 (1.00–3.29)
Hypertension	1.89*** (1.32–2.72)	1.26 (.86–1.86)
Diabetes	1.68** (1.16–2.42)	1.47* (1.00–2.16)
Ischemic heart disease	2.29** (1.39–3.76)	1.45 (.86–2.44)
Chronic heart failure	1.99 (.99–4.00)	1.25 (.61–2.59)
Stroke	1.33 (.74–2.40)	0.89 (.48–1.65)
Chronic kidney disease	2.55*** (1.62–4.01)	1.86* (1.15–3.00)
Dementia	2.67*** (1.58–4.50)	1.32 (.75–2.32)
DVT/PE (previous)	2.9 (.64–13.08)	3.07 (.65–14.40)
Atrial fibrillation	1.68* (1.01–2.80)	1.25 (.73–2.13)
COPD	1.07 (.47–2.44)	0.78 (.34–1.81)
Asthma	0.44* (.21–.93)	0.42* (.19–.91)
Liver disease (noncirrhotic)	0.77 (.38–1.58)	0.99 (.47–2.11)
Liver disease (cirrhotic)	3.08 (.96–9.84)	3.19 (.95–10.76)
Solid malignant tumor	1.21 (.68–2.16)	0.74 (0.40–1.35)
Hematologic malignancy	1.29 (.30–5.45)	1.24 (0.28–5.44)
HIV/AIDS	0.85 (.16–4.45)	1.32 (0.24–7.36)
	Unadjusted	Adjusted^a^ Model
Clinical observations on admission		
Fever	1.83** (1.25–2.67)	1.82** (1.18–2.80)
Respiratory rate		
<20 (intercept)	…	…
20–29	1.2 (.77–1.85)	…
≥30	2.18** (1.30–3.67)	…
SaO_2_		
≥95% on room air (intercept)		…
<95% on room air	2.14* (1.17–3.93)	1.82 (.96–3.47)
≥95% on FiO_2_ <60%	1.96* (1.14–3.35)	1.88* (1.05–3.37)
<95% on FiO_2_ <60%	2.77** (1.42–5.39)	2.67** (1.31–5.45)
Any on FiO_2_ ≥60%	5.41*** (3.17–9.25)	4.25*** (2.36–7.64)
MAP, mm Hg		
70–99 (intercept)	…	…
<70	1.96 (.96–4.00)	…
≥100	0.98 (.66–1.45)	…
Pulse		
60–99 (intercept)	…	…
<60	0.72 (.14–3.61)	…
≥100	1.02 (.71–1.48)	…
NEWS-2 ∆		
<4 (intercept)	…	…
4–6	1.34 (.83–2.15)	…
≥7	3.79*** (2.40–6.00)	…
Laboratory investigations on admission		
Hemoglobin		
≥130 (intercept)	…	…
100–129	2.21* (1.08–4.53)	…
<100	1.22 (.83–1.78)	…
White cell count		
4.2–10.5 (intercept)	…	…
<4.2	0.94 (.50–1.77)	0.78 (.38–1.59)
≥10.6	1.99** (1.24–3.19)	2.35** (1.35–4.11)
Lymphocytes		
1.1–3.4 (intercept)	…	…
<0.5	2.67** (1.40–5.10)	…
0.5–1.0	1.60* (1.06–2.40)	…
≥3.5	1.04 (.20–5.35)	…
Platelets (numeric)	1.00** (1.00–1.01)	1.01*** (1.00–1.01)
Albumin (numeric)	1.09*** (1.04–1.12)	1.06*** (1.02–1.09)
Bilirubin (numeric)	1.01 (.99–1.03)	…
ALP (numeric)	1.00 (1.00–1.00)	…
Creatinine		
<125 (intercept)	…	…
≥125	2.84 *** (1.91–4.22)	…
eGFR		
≥90 (intercept)	…	
60–89	3.25*** (1.79–5.89)	2.65** (1.47–4.78)
30–59	4.70*** (2.51–8.78)	3.78*** (2.03–7.04)
<30	8.57*** (4.43–16.57)	5.14*** (2.65–9.97)
Glucose		
3.7–5.1 (intercept)	…	…
<3.7	4.73 (.39–57.70)	…
≥5.2	1.14 (.55–2.38)	…
Missing	0.94 (.41–2.14)	…
CRP		
<10 (intercept)	…	…
10–99	3.67* (1.08–12.40)	…
≥100	7.15** (2.14–23.90)	…
Missing	11.00** (2.59–46.76)	…
CXR		
Normal or non–COVID-19 findings (intercept)	…	…
Mild	0.85 (.42–1.72)	…
Moderate/severe	1.54 (.93–2.54)	…
Missing	1.14 (.62–2.11)	…

Data are presented as odds ratios (95% confidence intervals).

Abbreviations: ALP, alkaline phosphatase; COPD, chronic obstructive pulmonary disease; COVID-19, coronavirus disease 2019; CRP, C-reactive protein; CXR, chest radiograph; DVT/PE, deep vein thrombosis/pulmonary embolism; eGFR, estimated glomerular filtration rate; FiO_2_, fraction of inspired oxygen; HIV, human immunodeficiency virus; IU, international units; MAP, mean arterial pressure; NEWS-2, New Early Warning Score; SaO_2_, oxygen saturation.

^a^Adjusted regression model for clinical and laboratory observations on admission. Only variables that remained statistically significant are shown. For full model selection process, see Supplementary Table 5. Final model’s area under the receiver operating characteristic curve = 0.77. NEWS-2 ∆ was not included in adjusted model, as this is already a measure of patient severity based on clinical observations (ie, consciousness, respiratory rate, SaO_2_, pulse, systolic blood pressure, supplementary oxygen, and temperature).

*P < .05.

**P < .01.

***P < .001.

Also in unadjusted regression, thrombocytopenia (increase: OR, 1.00 [95% CI 1.00–1.01], per 10^9^/L decrease), lymphopenia <0.5 × 10^9^ cells/L (OR, 2.67 [95% CI, 1.41–5.210]), leukocytosis ≥10.6 × 10^9^ cells/L (OR, 1.99 [95% CI, 1.24–3.19]), eGFR <30 mL/minute/1.73 m^2^ (OR, 8.57 [95% CI, 4.43–16.57]), low albumin (increase: OR, 1.09 [95% CI, 1.04–1.14], per g/dL decrease), and C-reactive protein ≥100 mg/dL (OR, 7.22 [95% CI, 2.16–24.13]) were associated with increased probability of death.

Hypertension, diabetes, ischemic heart disease, atrial fibrillation, chronic kidney disease, and dementia had an unadjusted association with mortality (Table 2). However, when accounting for age, only diabetes (adjusted OR [aOR], 1.47 [95% CI, 1.00–2.16]) and chronic kidney disease (aOR, 1.86 [95% CI, 1.15–3.00]) remained statistically significant. Asthma appeared to be a protective factor for death both in unadjusted regression and after adjusting for age (aOR, 0.42 [95% CI, .19–.91]) (Table 2).

In adjusted logistic regression, clinical and laboratory observations with an increased odds ratio of mortality that remained statistically significant were fever (≥38°C) on admission, having a SaO_2_ <95% while on supplementary oxygen or if >60% FiO_2_ was administered on admission, elevated white cell count, thrombocytopenia (continuous), hypoalbuminemia (continuous), and a reduced eGFR (Table 2) (area under the receiver operating curve [AUROC] = 0.77) (Supplementary Table 5).

### Outcomes by Ethnicity

Two hundred thirty-five (38%) patients were of white background, 133 (22%) were black, 94 (15%) Asian, 17 (3%) “other,” and 135 (22%) of unknown ethnicity. Overall, neither the intermediate (requirement of FiO_2_ ≥60% or invasive ventilation) nor final outcomes (death or discharged alive) were significantly different between ethnic groups (Table 3). However, white patients were significantly older, with an average age of 70.6 years (compared to 63.6 years for black, 66.4 Asian, and 54.8 for “other” ethnicity) (*P* < .01) and also had a higher burden of pre-existing comorbidities as defined by the Elixhauser score (*P* = .02) (Table 3 and Supplementary Figure 4).

**Table 3.** Clinical Characteristics by Ethnicity and Logistic Regression of Odds of Death

Characteristic	White (n = 235)	Black (n = 133)	Asian (n = 94)	Other (n = 17)	Missing (n = 135)	*P* Value
Male sex	146 (62.13)	76 (57.14)	60 (63.83)	12 (70.59)	88 (65.19)	.63
Mean age, y (SD)	70.52 (15.69)	63.58 (19.08)	66.4 (15.88)	54.82 (15.25)	60.7 (18.11)	<.01
Mean Elixhauser score (SD)	6.29 (7.25)	5.23 (6.85)	6.63 (7.15)	2.53 (4.37)	2.99 (5.12)	<.01
Mean NEWS-2 score (SD)	5.09 (3.2)	4.93 (3.04)	4.74 (3.31)	5.41 (2.67)	5.39 (2.83)	.28
Mean days to admission (SD)	6.99 (5.75)	7.33 (5.98)	6.15 (4.82)	7.29 (6.47)	8.16 (5.08)	.49
Outcomes						
FiO_2_ ≥60%	100 (42.55)	61 (45.86)	43 (45.74)	5 (29.41)	70 (51.85)	.31
Admitted to ICU	23 (9.79)	20 (15.04)	15 (15.96)	2 (11.76)	27 (20.00)	.10
Received IVS	22 (9.36)	18 (13.53)	14 (14.89)	2 (11.76)	24 (17.78)	.21
Died in hospital	67 (28.51)	41 (30.83)	30 (31.91)	3 (17.65)	37 (27.41)	.76
Discharged alive	149 (63.40)	78 (58.65)	54 (57.45)	14 (82.35)	86 (63.70)	.31
Pending outcome	19 (8.09)	14 (10.53)	10 (10.64)	0 (0.00)	12 (8.89)	.62
Comorbidities						
Any comorbidity	179 (76.17)	112 (84.21)	78 (82.98)	11 (64.71)	100 (74.07)	.10
Ischemic heart disease	33 (14.04)	16 (12.03)	18 (19.15)	0 (0.00)	13 (9.63)	.12
Chronic heart failure	19 (8.09)	11 (8.27)	1 (1.06)	0 (0.00)	6 (4.44)	.07
Hypertension	97 (41.28)	73 (54.89)	40 (42.55)	7 (41.18)	69 (51.11)	.08
Hyperlipidemia	57 (24.26)	32 (24.06)	27 (28.72)	2 (11.76)	33 (24.44)	.66
Diabetes	61 (25.96)	63 (47.37)	41 (43.62)	4 (23.53)	47 (34.81)	<.01
CKD	39 (16.60)	26 (19.55)	26 (27.66)	0 (0.00)	11 (8.15)	<.01
Peripheral vascular disease	11 (4.68)	3 (2.26)	0 (0.00)	0 (0.00)	4 (2.96)	.19
Stroke	28 (11.91)	13 (9.77)	11 (11.70)	1 (5.88)	4 (2.96)	.05
Atrial fibrillation	43 (18.30)	10 (7.52)	11 (11.70)	2 (11.76)	9 (6.67)	<.01
DVT/PE history	4 (1.70)	2 (1.50)	0 (0.00)	0 (0.00)	1 (0.74)	.68
Hemiplegia	6 (2.55)	0 (0.00)	0 (0.00)	0 (0.00)	0 (0.00)	.04
Dementia	42 (17.87)	9 (6.77)	10 (10.64)	0 (0.00)	9 (6.67)	<.01
Asthma	18 (7.66)	14 (10.53)	8 (8.51)	3 (17.65)	13 (9.63)	.65
COPD	22 (9.36)	3 (2.26)	3 (3.19)	0 (0.00)	2 (1.48)	<.01
Connective tissue disease	4 (1.70)	3 (2.26)	1 (1.06)	0 (0.00)	1 (0.74)	.82
Peptic ulcer	8 (3.40)	2 (1.50)	1 (1.06)	0 (0.00)	3 (2.22)	.61
Liver (noncirrhotic)	14 (5.96)	12 (9.02)	10 (10.64)	3 (17.65)	5 (3.70)	.09
Liver (cirrhotic)	2 (0.85)	4 (3.01)	6 (6.38)	0 (0.00)	1 (0.74)	.02
Solid tumor	33 (14.04)	9 (6.77)	8 (8.51)	1 (5.88)	8 (5.93)	.06
Hematologic tumor	4 (1.70)	1 (0.75)	1 (1.06)	0 (0.00)	2 (1.48)	.92
HIV/AIDS	1 (0.43)	7 (5.26)	1 (1.06)	0 (0.00)	0 (0.00)	<.01
Logistic regression of odds of death by ethnicity						
Unadjusted, OR (95% CI)	Intercept	1.17 (.73–1.88)	1.24 (.73–2.10)	0.48 (.13–1.71)	0.96 (.59–1.55)	
Adjusted^a^ model, OR (95% CI)	Intercept	1.85* (1.06–3.24)	1.78 (.97–3.29)	0.95 (.24–3.80)	1.63 (.93–2.84)	

Data are presented as no. (%) unless otherwise indicated.

Abbreviations: CKD, chronic kidney disease; COPD, chronic obstructive pulmonary disease; DVT/PE, deep vein thrombosis/pulmonary embolism; FiO_2_, fraction of inspired oxygen; NEWS-2, New Early Warning Score; ICU, intensive care unit; HIV, human immunodeficiency virus; IVS, invasive ventilation support; SD, standard deviation.

^a^Adjusted regression model for age, male sex, Elixhauser score, and NEWS-2 differences by ethnic groups. Additional logistic regression models adjusted for any comorbidity, diabetes, and CKD were also carried out, but were inferior predictors compared to the selected model. COPD, cirrhotic liver disease, and HIV/AIDS were not used in logistic regression models, as they had low n values for ethnic groups.

*P < .05.

**Figure 2.**
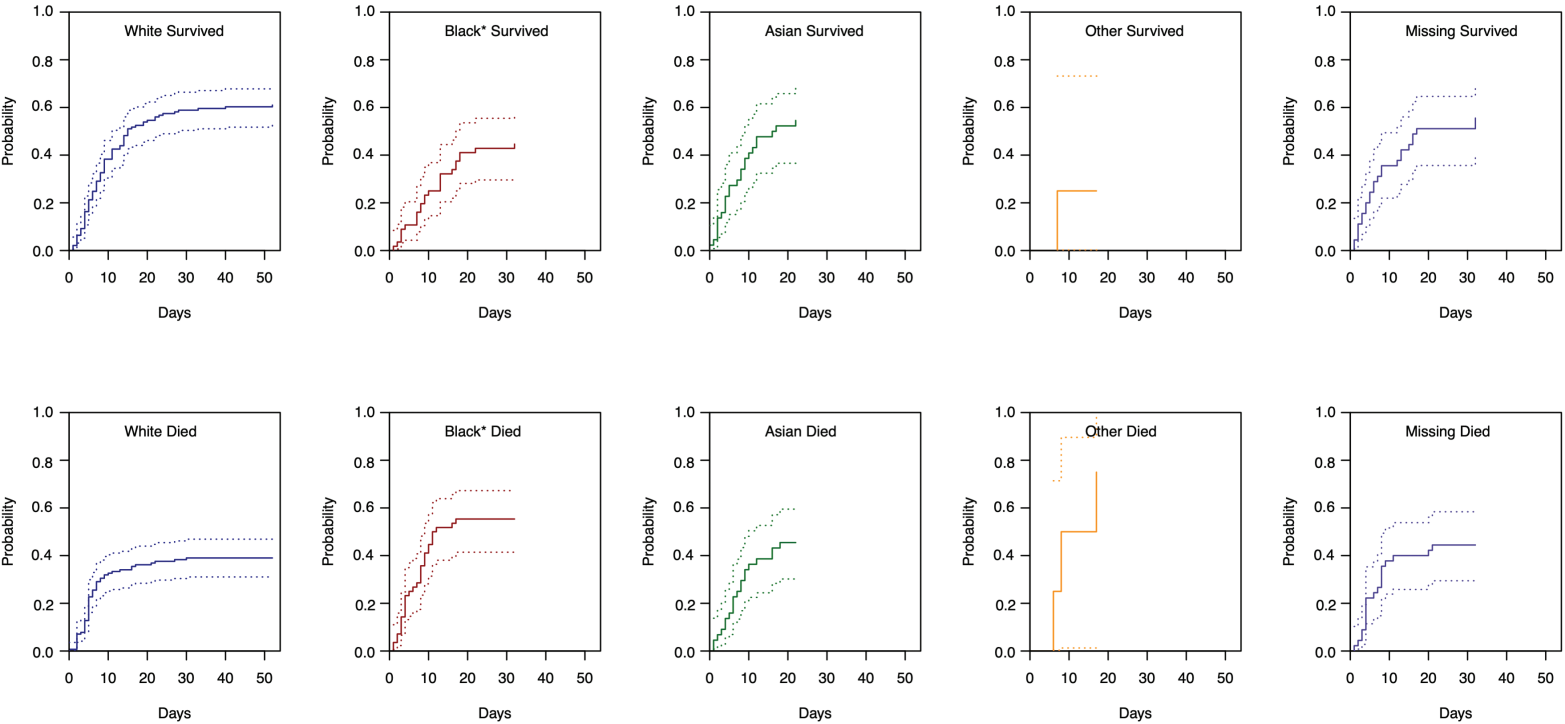
Cumulative probability of hospitalization outcome among those older than the median age of the cohort (ie, 69 years) by ethnic group. Calculated with Nelson-Aalen estimator. Note from text and Table 3 that white patients were significantly older than black, Asian, and other ethnic minority groups. *P < .05.

Despite their younger age composition and lower Elixhauser score, the severity of disease on presentation (NEWS-2) was similar between patients of BAME and white ethnicity (*P* = .28) (Table 3 and Supplementary Figure 4). Furthermore, when stratifying ethnic groups by the median age of the overall cohort (ie, 69 years), we found that the cumulative probability of death of black patients was in fact higher than that of their white counterparts (Figure 2).

We thus performed unadjusted and adjusted logistic regression analyses to assess the odds of death of BAME groups compared to white patients. The unadjusted odds of death did not differ across ethnic groups (Table 3 and Supplementary Table 4). However, when adjusting for age, sex, comorbidities, and NEWS-2 on admission, the odds of death of black compared to white patients was higher (aOR, 1.85 [95% CI, 1.06–3.24]; *P* = .04; AUROC = 0.78) (Table 3). If admission severity by NEWS-2 was removed from the regression model, the trend remained, albeit less strongly (aOR, 1.69 [95% CI, 1.00–2.86]; *P* = .049; AUROC = 0.73) (Supplementary Table 4). Clinically relevant, albeit marginally nonstatistically significant, trends were observed for Asian (aOR, 1.78 [95% CI, .97–3.29]; *P* = .06) patients and those of unknown ethnicity (Table 3). The proportion of patients with clinical and laboratory abnormalities on admission associated with increased odds of death also varied by ethnic group (Supplementary Figure 6). Notably, eGFR alterations (*P* < .01) and fever (*P* = .01) were more prevalent among BAME groups. The prevalence of the rest of the prognostic markers did not show significant variations by ethnicity.

## DISCUSSION

We present detailed demographic, clinical, and outcome data for 614 laboratory-confirmed SARS-CoV-2 cases at a large multiethnic London NHS Trust. Our study provides methodologically robust evidence that, beyond the widely reported factors associated with increased odds of COVID-19 mortality (age, sex, and severe hypoxemia on admission), thrombocytopenia, leukocytosis, hypoalbuminemia, and reduced eGFR are also significantly associated with increased in-hospital death. Furthermore, we present an in-depth analysis of the clinical association between ethnicity and patient outcomes during the COVID-19 epidemic in the UK, a current topic of high global health priority. In addition to an overrepresentation of BAME patients in our hospitalized COVID-19 cohort (when compared to historic admissions to ICHNT; see Supplementary Table 3), we find an association of increased odds of death among black (compared to white) patients, when adjusted for age, sex, burden of comorbidities, and severity of disease on admission.

Emerging evidence on the associations between ethnicity and COVID-19 outcomes has been conflicting. In the UK, reports from gray literature highlight a higher risk of death for BAME than for white patients, which has been partially explained by sociodemographic differences and pre-existing comorbidities [8, 11]. An Office for National Statistics report, however, relied on historical population census data (self-reported health indices) to determine these associations [11], and a preprint study focused on comorbidities without admission severity or other clinical observations [8]. Our study thus adds an important clinical perspective, using comprehensive granular patient-level data, which national datasets cannot often provide.

We find that the underlying profiles of the different ethnic groups varied, with black and “other” ethnic minority groups being younger, and Asian and white groups having a higher burden of pre-existing comorbidities. A high prevalence of undiagnosed comorbidities among BAME groups has been previously identified [20], although the significance of this in the current pandemic is yet unknown. We observed that after adjusting for potential confounders, such as age, sex, comorbidities, and severity of admission, black (compared to white) patients were at increased odds of death. This clinically relevant trend was also seen for Asian patients, although it was marginally nonstatistically significant. Interestingly, another recent UK study found ethnicity to be 1 of 8 covariates predicting ICU admission but not mortality [21]. In that study, though, the majority of nonwhite patients were black, whereas our study had high representation of both black and Asian patients. Together, our findings and those from other emerging studies suggest that important biological factors may be potentially driving differences in COVID-19 hospitalization outcomes by ethnicity, which cannot solely be explained by socioeconomic differences.

The symptom profile in our cohort was largely consistent with previous studies [4–6, 12, 21]. However, both in our study and others from the UK [12], a much higher proportion of patients reported shortness of breath on admission compared with what has been reported in China (65%–67% vs 25%) [22]. Furthermore, a higher proportion of our cohort showed abnormal inflammatory markers and thrombocytopenia. These discrepancies could reflect differing thresholds and criteria for hospitalization between the UK and other countries. High mortality rates have been seen in many settings, for example, 26% among all admissions in an Italian study and 88% for mechanically ventilated patients in a study from the United States [6, 23]. We found a crude mortality rate of 29% overall and of 52% among ventilated patients, albeit 55 patients in our study remained hospitalized at the time of censoring. The extent to whether intercountry variations in clinical practice and capacity restraints affects in-hospital mortality is currently unknown and warrants further investigation.

A key strength of our study is that we have systematically identified associations between demographic and clinical factors associated with outcomes in a cohort of >600 patients through multiple regression, survival and competing risks analyses. Previous studies have focused on descriptive statistics [4–6, 12, 22], with few robustly correcting for covariates in their evaluation of outcomes [21].

However, limitations to our study should be acknowledged. First, although we attempted to be as comprehensive as possible, the retrospective nature of this study limited the availability of data. Missing values, including of key variables such as BMI, ethnicity, and pre-existing comorbidities, could be possibly related to the severity of disease on admission and we may have thus missed important associations with mortality. We tried to overcome these data limitations as thoroughly as possible by cross-checking medical clerking on admission and discharge, with patients’ referral letters and historic records, where available. Also, many of the nonroutine laboratory tests (including D-dimer, brain natriuretic peptide, and troponin) were only introduced systematically at ICHNT a few weeks into the pandemic. Although descriptive statistics suggest there are differences in the prevalence of these variables by patient outcomes, they were excluded from the multiple logistic regression given incomplete recording. Strengthening of data recording has been implemented in our hospitals. This, together with standardized laboratory investigation protocols, will improve future observational studies, as well as the monitoring of patient outcomes in intervention trials.

Finally, this study did not consider the characteristics and outcomes of those who were hospitalized with clinical features of COVID-19 but were RT-PCR swab negative and those who might have had hospital-acquired SARS-CoV-2 infection. The profiles of these patients and whether their outcomes are different to the patients presented here require further research. Furthermore, we could not quantify the impact of changes in clinical practice over the time of our study. However, only a few patients were enrolled in clinical trials early in the pandemic, so changing clinical practices are unlikely to impact on our study findings.

## CONCLUSIONS

This is one of the first studies in the UK to analyze the associations between demographic and clinical characteristics of COVID-19 patients and their hospitalization outcomes by ethnicity. We have employed robust methodological analyses to account for the effect of patients with uncompleted outcomes. The admission characteristics with the strongest association with increased odds of death were fever, severe hypoxemia, thrombocytopenia, leukocytosis, hypoalbuminemia, and a reduced eGFR. BAME groups were overrepresented in our study population, compared with historical admissions to our hospital trust. After accounting for age, comorbidity profile, and severity of disease on admission, patients of black and Asian ethnicity appear to have the worst hospitalization outcomes. Research is urgently needed into complex multifactorial interactions underpinning differences in COVID-19 disease outcomes by ethnicity. Such analyses would have a vital role in informing changes in clinical and public health practice locally and abroad.

## Supplementary Data

Supplementary materials are available at *Clinical Infectious Diseases* online. Consisting of data provided by the authors to benefit the reader, the posted materials are not copyedited and are the sole responsibility of the authors, so questions or comments should be addressed to the corresponding author.

ciaa1091_suppl_Supplementary_Material

## Notes

***Author contributions.*** S. N., K. H., T. B. H., and P.P.-G. conceived the study. S. N. and P.P.-G. wrote the first manuscript draft. P.P.-G., M. D. K., and M. V. analyzed the data. P. A. and P. J. W. provided methodological expertise. S. N., P.P.-G., M. R. T., P. A., P. J. W., K. H., and T. B. H. interpreted the data. S. N., P.P.-G., A. D., S. M., R. F., P. C., P. M., R. J., C. H., and A. S. collected the data. S. N., P.P.-G., M. R. T., and G. C. provided clinical expertise. All authors reviewed and edited the manuscript for scientific content.

***Acknowledgments.*** The authors acknowledge the Abdul Latif Jameel Institute for Disease and Emergency Analytics (J-IDEA), the Imperial Biomedical Research Centre, and the Medical Research Council (MRC) Centre for Global Infectious Disease Analysis for providing the platforms for multicollaborative projects such as this one. The authors would like to thank all patients at Imperial College Healthcare NHS Trust and its staff, who have been involved in the management of patients during this pandemic, in particular, Julian Redhead, Tim Orchard, Georgie Russell, Claire Ross, Susie Bloch, Ernie Wong, Simon Ashworth, and Carlos Gomez. We would also like to thank Jonathan Clarke, Luca Mercuri, Ben Glampson, and Dimitri Papadimiriou for providing data support for the analysis.

***Disclaimer.*** The study sponsors had no role in study design, data collection, data analysis, data interpretation, report writing, or decision to submit for publication. The views expressed are those of the authors and not necessarily those of the funders. The corresponding author had full access to all study data and had final responsibility for the decision to submit for publication.

***Financial support.*** We acknowledge funding from the MRC Centre for Global Infectious Disease Analysis (MR/R015600/1); this award is jointly funded by the UK MRC and the UK Department for International Development (DFID) under the MRC/DFID Concordat agreement and is also part of the EDCTP2 program supported by the European Union. K. H. acknowledges joint center funding from J-IDEA.

***Potential conflicts of interest.*** P. A. reports a research grant from Dr Foster Ltd (a wholly owned subsidiary of Telstra Health). All other authors report no potential conflicts. All authors have submitted the ICMJE Form for Disclosure of Potential Conflicts of Interest. Conflicts that the editors consider relevant to the content of the manuscript have been disclosed.
