# Supplementary material for: Clinical Characteristics and Predictors of Outcomes of Hospitalized Patients With Coronavirus Disease 2019 in a Multiethnic London National Health Service Trust: A Retrospective Cohort Study
Source: Clin Infect Dis. 2020 Aug 7;73(11):e4047–57. doi: 10.1093/cid/ciaa1091 (PMC7454410; doi:10.1093/cid/ciaa1091)
Supplement: ciaa1091_suppl_Supplementary_Material [file ciaa1091_suppl_supplementary_material.docx]

**Supplementary Appendix - Clinical characteristics and predictors of outcomes of hospitalized patients with COVID-19 in a London NHS Trust: a retrospective cohort study**

This supplementary appendix presents additional figures and tables to fully characterize the cohort of COVID-19 patients at hospitalized at Imperial College Healthcare NHS Trust between the 25^th^ of February and 5^th^ of April 2020.

For radiologic description of finding, we used the classification system proposed by the British Society of Thoracic Imaging,[1] as follows: (i) radiological findings (CVCX0 = Normal, CVCX1 = Classic, CVCX2 = Indeterminate, CVCX3 = Non-COVID-19) and (ii) quantification of radiographic disease severity (mild, moderate, severe).13 As CT scans were not systematically performed for all COVID-19 patients in this clinical setting, they were excluded from this analysis.

**Equation S1. Cumulative transition hazard and survival function defined by the Nelson-Aeler (a) and Kaplan-Meier (b) estimators.**

$$a) Cumulative transition hazard \left( t \right)= \sum_{k=1}^{K} \frac{number observed \longrightarrow transitions at t_{k}}{numer at risk just prior to t_{k}}$$

$$b) Survival \left( t \right)= \prod_{t_{i}\leq t} (1- \frac{d_{i}}{n_{i}})$$

**Supplementary Table S1:** Mean (SD) of numeric clinical variables on admission

|  | **All (n = 615)** | **Discharged (dead) (n = 179)** | **Discharged (recovered)**  **(n = 381)** | **p value** |
| --- | --- | --- | --- | --- |
| SaO2 | 94.72 (4.93) | 93.41 (5.28) | 95.63 (4.1) | <0.01 |
| Temperature | 37.45 (1.1) | 37.52 (1.09) | 37.41 (1.09) | 0.27 |
| Respiratory rate | 24.24 (7.59) | 25.18 (7.45) | 23.51 (7.47) | <0.01 |
| Pulse | 94.8 (19.3) | 95.96 (19.03) | 93.99 (19.88) | 0.26 |
| MAP | 94.53 (16.88) | 92.81 (19.57) | 95.03 (15.44) | 0.18 |
| NEWS-2 | 5.08 (3.09) | 6.23 (3.21) | 4.43 (2.77) | <0.01 |
| BMI | 28.85 (7.23) | 29.47 (8.23) | 28.62 (6.76) | 0.38 |
| Haemoglobin | 132.6 (19.58) | 129.57 (20.48) | 133.47 (19.01) | 0.03 |
| White blood cells | 7.93 (4.29) | 8.33 (4.01) | 7.59 (4.32) | 0.02 |
| Lymphocytes | 1.13 (1.24) | 1.01 (1.35) | 1.13 (0.84) | <0.01 |
| Platelets | 212.75 (91.04) | 194.91 (87.73) | 221.97 (91.38) | <0.01 |
| Creatinine | 138.6 (161.26) | 184.13 (207.11) | 115.75 (130.58) | <0.01 |
| Urea | 8.7 (7.96) | 11.5 (8.58) | 7.49 (7.69) | <0.01 |
| GFR (MDRD) | 68.5 (47) | 50.74 (27.37) | 68.29 (24.89) | <0.01 |
| Albumin | 31.07 (5.12) | 29.83 (5.03) | 31.96 (5.04) | <0.01 |
| Total bilirubin | 13.44 (11.66) | 14.29 (10.17) | 12.94 (12.58) | 0.09 |
| ALT | 46.38 (108.46) | 54.83 (179.51) | 43.12 (65.12) | 0.22 |
| ALP | 94.81 (63.09) | 99.25 (49.59) | 93.38 (69.47) | 0.05 |
| PT | 13.8 (1.7) | 16.49 (11.38) | 16.31 (16.7) | 0.08 |
| Lactate | 1.73 (1.56) | 2.08 (2.19) | 1.56 (1.18) | <0.01 |
| Glucose | 9.1 (5.58) | 9.38 (5.75) | 8.6 (4.67) | 0.19 |
| CRP | 103.65 (133.27) | 151.66 (102.78) | 106.3 (91.76) | <0.01 |
| D-Dimer | 2506.73 (3942.01) | 2874.63 (4103.94) | 2283.74 (3862.81) | 0.04 |
| LDH | 505.21 (434.52) | 623.8 (680.95) | 439.86 (273.7) | <0.01 |
| Troponin | 14 (35.5) | 252.69 (904.56) | 86.8 (914.33) | <0.01 |
| CK | 541.34 (1355.61) | 695.31 (1173.64) | 497.39 (1532.79) | <0.01 |
| Cortisol (random) | 802.14 (572.51) | 904 (544.45) | 719.29 (579.67) | 0.02 |
| BNP | 37 (98) | 433.29 (1569.13) | 105.9 (277.8) | <0.01 |
| Ferritin | 2010.93 (9717.62) | 1672.31 (2841.14) | 2236.05 (12487.42) | 0.50 |

**Supplementary Table S2:** Comorbidities and outcomes by age

*Abbreviations: 95% CI, 95% confidence interval; COPD, chronic obstructive pulmonary disease; DVT/PE, deep vein thrombosis / pulmonary embolism; HIV/AIDS, human immunodeficiency virus / acquired immunodeficiency syndrome; HDU/ITU, high dependency unit / intensive treatment unit; OR, odds ratio*

|  | **<18**  **(n = 4)** | **18-29**  **(n = 9)** | **30-39**  **(n = 34)** | **40-49**  **(n = 45)** | **50-59**  **(n = 120)** | **60-69**  **(n = 93)** | **70-79**  **(n = 139)** | **80+**  **(n = 171)** |
| --- | --- | --- | --- | --- | --- | --- | --- | --- |
| **Comorbidities (%)** |  |  |  |  |  |  |  |  |
| Any comorbidity | 0 (0%) | 3 (33.3%) | 11 (32.4%) | 18 (40%) | 70 (58.3%) | 52 (55.9%) | 96 (69.1%) | 133 (77.8%) |
| Hypertension | 0 (0%) | 0 (0%) | 3 (8.8%) | 7 (15.6%) | 37 (30.8%) | 35 (37.6%) | 56 (40.3%) | 89 (52%) |
| Diabetes | 0 (0%) | 0 (0%) | 5 (14.7%) | 6 (13.3%) | 37 (30.8%) | 25 (26.9%) | 47 (33.8%) | 50 (29.2%) |
| Ischaemic heart disease | 0 (0%) | 0 (0%) | 0 (0%) | 0 (0%) | 4 (3.3%) | 6 (6.5%) | 22 (15.8%) | 29 (17%) |
| Chronic heart failure | 0 (0%) | 0 (0%) | 0 (0%) | 1 (2.2%) | 0 (0%) | 5 (5.4%) | 7 (5%) | 14 (8.2%) |
| Stroke | 0 (0%) | 0 (0%) | 0 (0%) | 0 (0%) | 7 (5.8%) | 3 (3.2%) | 12 (8.6%) | 22 (12.9%) |
| Chronic kidney disease | 0 (0%) | 0 (0%) | 2 (5.9%) | 3 (6.7%) | 12 (10%) | 5 (5.4%) | 23 (16.5%) | 38 (22.2%) |
| Dementia | 0 (0%) | 0 (0%) | 0 (0%) | 0 (0%) | 1 (0.8%) | 1 (1.1%) | 11 (7.9%) | 38 (22.2%) |
| DVT/PE (previous) | 0 (0%) | 0 (0%) | 0 (0%) | 0 (0%) | 1 (0.8%) | 3 (3.2%) | 1 (0.7%) | 2 (1.2%) |
| Atrial fibrillation | 0 (0%) | 0 (0%) | 1 (2.9%) | 2 (4.4%) | 8 (6.7%) | 11 (11.8%) | 19 (13.7%) | 30 (17.5%) |
| COPD | 0 (0%) | 0 (0%) | 0 (0%) | 1 (2.2%) | 2 (1.7%) | 4 (4.3%) | 9 (6.5%) | 7 (4.1%) |
| Asthma | 0 (0%) | 2 (22.2%) | 2 (5.9%) | 7 (15.6%) | 9 (7.5%) | 5 (5.4%) | 8 (5.8%) | 14 (8.2%) |
| Liver disease (non-cirrhotic) | 0 (0%) | 1 (11.1%) | 3 (8.8%) | 4 (8.9%) | 7 (5.8%) | 11 (11.8%) | 10 (7.2%) | 6 (3.5%) |
| Liver disease (cirrhotic) | 0 (0%) | 0 (0%) | 0 (0%) | 0 (0%) | 5 (4.2%) | 0 (0%) | 4 (2.9%) | 2 (1.2%) |
| Solid malignant tumour | 0 (0%) | 0 (0%) | 0 (0%) | 0 (0%) | 2 (1.7%) | 6 (6.5%) | 17 (12.2%) | 21 (12.3%) |
| Haematologic malignancy | 0 (0%) | 0 (0%) | 0 (0%) | 0 (0%) | 0 (0%) | 2 (2.2%) | 3 (2.2%) | 1 (0.6%) |
| HIV/AIDS | 0 (0%) | 0 (0%) | 1 (2.9%) | 1 (2.2%) | 2 (1.7%) | 1 (1.1%) | 2 (1.4%) | 0 (0%) |
| **Outcomes (%)** |  |  |  |  |  |  |  |  |
| Required FiO2 > 0.6 | 0 (0) | 1 (11.1%) | 7 (20.6%) | 21 (46.7%) | 61 (50.8%) | 46 (49.5%) | 64 (46%) | 80 (46.8%) |
| Admitted to ITU | 0 (0%) | 0 (0%) | 6 (17.6%) | 13 (28.9%) | 30 (25%) | 25 (26.9%) | 10 (7.2%) | 3 (1.8%) |
| Received IVS | 0 (0%) | 0 (0%) | 6 (17.6%) | 13 (28.9%) | 27 (22.5%) | 24 (25.8%) | 8 (5.8%) | 2 (1.2%) |
| Died in hospital | 0 (0%) | 1 (11.1%) | 1 (2.9%) | 1 (2.2%) | 22 (18.3%) | 24 (25.8%) | 50 (36%) | 80 (46.8%) |

**Supplementary Table S3:** Ethnicity breakdown compared with previous emergency admissions at ICHNT

| **Ethnicity** | **ICHNT COVID-19 2020** | **Emergency admissions of ≥18-year-olds ICHNT 2018/19** |
| --- | --- | --- |
| White | 235 (38%) | 21,236 (45%) |
| Asian | 133 (22%) | 4,182 (9%) |
| Black | 94 (15%) | 5,130 (11%) |
| Other | 17 (3%) | 7,936 (17%) |
| Unknown | 135 (22%) | 8,019 (18%) |

**Figure S1:** Daily new COVID-19 laboratory confirmed cases at ICHNT

**Figure S2**: Kaplan-Meier curves for length of hospital stay and time to clinical deterioration, defined as requiring FiO2 ≥60%, receiving invasive ventilation or being admitted to ICU


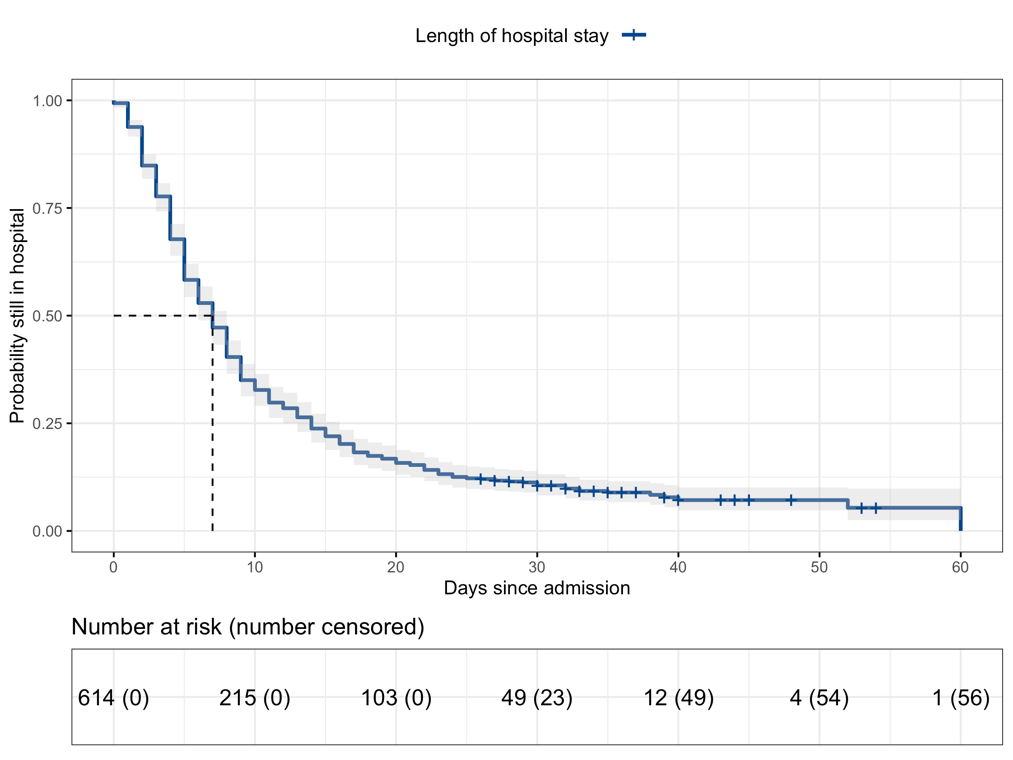

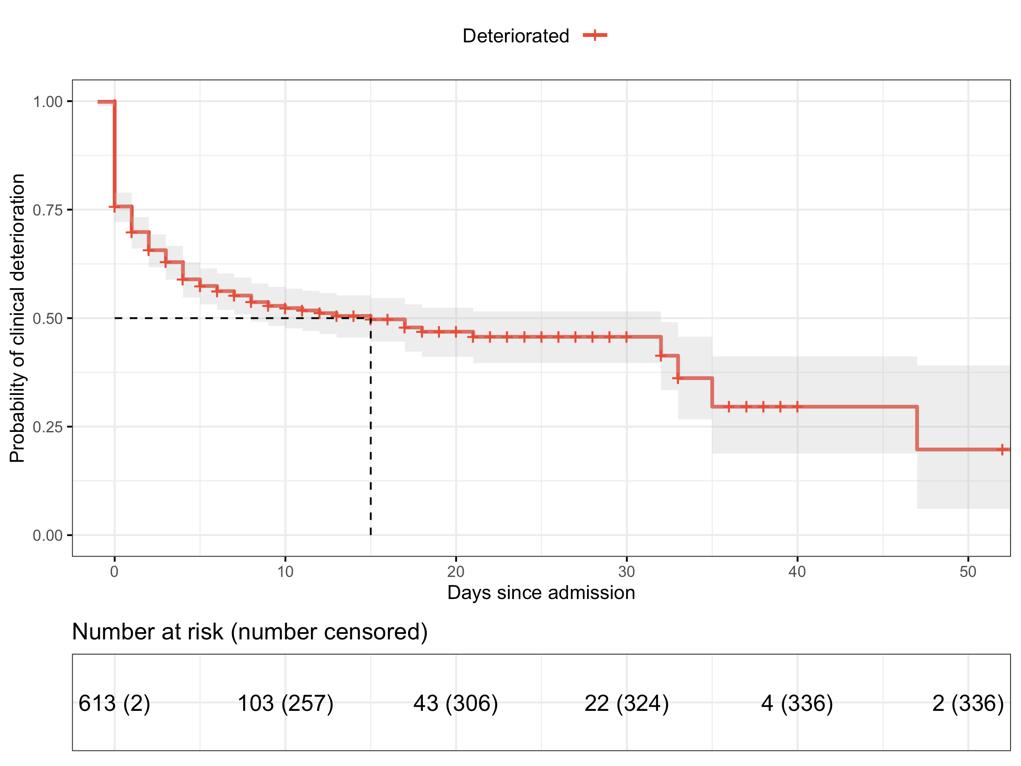


**Figure S3**: Cumulative incidence function of competing risks of hospitalisation outcomes, accounting for censoring, by age

**Figure S4:** Age, NEWS-2 and Elixhauser score by ethnic groups

**Figure S5:** Cumulative incidence function for hospitalization outcomes, accounting for censoring, by ethnic groups

**Figure S6:** Prevalence (n) of admission clinical and laboratory variables predictive of death by ethnic groups

*Combined prevalence (n) of either having a SaO2 <95% on FiO2 < 60% or any SaO2 whilst on FiO2 ≥ 60%. Note that for clarity, we present the prevalence (n) of having platelets < 130 x 109L and albumin < 25g/L, albeit these were treated as numeric in logistic regression.

Abbreviations: eGFR, estimated glomerular filtration rate; FiO2, oxygen inspiratory fraction; SaO2, oxygen saturation; WCC, white cell count.

**Supplementary Table S4:** Adjusted model selection for odds of death by ethnicity

*** p < 0·001; ** p < 0·01; * p < 0·05

*Abbreviations: AIC, Akaike information criterion; AUROC, area under the receiver operating curve; BIC, Bayesian information criterion; CKD, chronic kidney disease; NEWS-2, New Early Warning Score*

**Supplementary Table S5:** Adjusted model selection for odds of death by clinical and laboratory observations on admission

*** p < 0·001; ** p < 0·01; * p < 0·05

† The mathematical inverse for these values is reported for main manuscript and Table 2, as the intercept for numeric variables was the lowest value in the cohort (e.g. aOR for albumin the odds of death increase by 1/0.94 = 1.06 for every g decrease from the maximum value in the cohort)

*Abbreviations: ALP, alkaline phosphatase; AUROC, area under the receiver operating curve; AIC, Akaike information criterion; BIC, Bayesian information criterion; CRP, C-reactive protein; CXR, chest X-ray; FiO2, oxygen inspiratory fraction; GFR, glomerular filtration rate; Hb, hemoglobin; MAP, mean arterial pressure; SaO2, oxygen saturation; WCC, white cell count.*

**Supplementary Table S6:** STROBE Checklist. [2]

| Item | Comment |
| --- | --- |
| 1. Title and abstract | We have indicated the study as a retrospective cohort in the title. The abstract clarifies the corresponding methods followed. |
| 2. Background and rationale | This is the first study from the UK on the clinical, laboratory and ethnic characteristics of hospitalization outcomes of patients with COVID-19. |
| 3. Objectives | 1. Describe baseline characteristics and outcomes for patients hospitalized with laboratory-confirmed SARS-CoV-2 infection in a large London hospital trust since the start of the pandemic, 2. Evaluate demographic and clinical factors associated with outcomes through logistic regression, and 3. Evaluate the proportion of patients hospitalized at ICHNT for COVID-19 who are from BAME groups and evaluate whether ethnicity is associated with poorer outcomes |
| 4. Study design | The retrospective cohort nature is highlighted in the title, abstract and first sentence of the methods section |
| 5. Setting | Imperial College Healthcare NHS Trust, comprised of three large hospitals in London (St Mary’s, Hammersmith and Charing Cross), from February 25 to April 5, 2020, and outcomes as of April 19, 2020. |
| 6. Participants | All consecutive patients with RT-PCR-confirmed COVID-19 disease requiring hospitalization. We excluded patients without COVID-19 symptoms on admission, patients with clinical suspicion of COVID-19 but with a negative RT-PCR, those who did not require hospitalization, transfers from other NHS Trusts and non-index (i.e. not the first positive hospitalization with a positive RT-PCR) hospitalization episodes. |
| 7. Variables | Outcomes of interest are clearly identified as whether the patient was discharged alive or died in hospital and clinical deterioration, defined as either requiring a 60% or greater concentration of supplementary oxygen to maintain oxygen saturation (SaO2) > 94%, invasive ventilation or admission to ITU. Variables used as explanatory are clearly documented (including units of measurement) in Table 1. |
| 8. Data sources | Individual patient electronic medical records (Cerner Hospital and Healthcare Systems and ICU computer records systems) were used to extract data. |
| 9. Bias | For completeness, ethnicity data for those with a record of ‘other ethnic background’ was checked against London Ambulance Service records, previous clinic letters and GP referral letters and reclassified as appropriate. To correctly identify variables as those present at the time of patient admission, serum creatinine, urea, glucose and lactate were only included if the blood test was performed within 4 hours of admission. All other blood tests and chest radiography were included if performed within 24 hours of admission. |
| 10. Study size | A description of the study size is provided in the methods and results sections, as well as in Figure 1. |
| 11. Quantitative variables | Numeric variables (e.g. age, hemoglobin, creatinine, etc.) were treated as continuous if their density distribution plot showed a normal distributed in either the natural or logarithmic scale, else they were coded into categorical. |
| 12. Statistical methods | We used standard *χ^2^*, t-student or Wilcoxon rank sum tests, as appropriate, to identify differences in the prevalence of covariates at admission among patients with a completed outcome. Variables with a *p-*value below 0·157 in univariate analysis were selected for multivariate analysis. In the multivariate models, variables were considered statistically significant with a *p*-value < 0·05 and thus kept as final candidates in the prediction model. Variables with more than 20% missing values were excluded from regression analysis. We evaluated time from hospital admission to clinical deterioration and final outcome outcomes accounting for competing risks and right-censored using the Nelson-Aelen and Kaplan-Meier estimators, respectively. The effect of selected covariates on the hazard of the clinical deterioration and final outcome (e.g. age) was assessed using Cox Proportional-Hazard models. The proportional hazards assumptions were evaluated using Schoenfeld’s residuals. |
| 13. Participants | We provide a diagram of patient participation in Figure 1. |
| 14. Descriptive data | The study population’s characteristics are summarized in the first section of the results section and in Tables 1 and 2. Missing data levels above 20% are identified in Table 2B (*). Cohort follow-up is summarized in Figure 2A, including number of patients under observation and censored at each time step. |
| 15. Outcome data | We report probability of discharge and cumulative probability of outcomes in Figures 2A, 2B and 3B. Time-dependent outcomes are further commented on in the results section of the manuscript, as appropriate. |
| 16. Main results | Unadjusted and adjusted estimates are clearly summarized in Tables 3 and 5 |
| 17. Other analyses | Subgroup analyses, by age and ethnicity, are summarized in Table 5 and Figures 2B and 3B |
| 18. Key results | In our study population, the admission characteristics associated with increased odds of hospital mortality on multivariate regression included severe hypoxia, low platelet count, elevated bilirubin and reduced eGFR. Our results suggest BAME groups are over-represented amongst those hospitalized at ICHNT for COVID-19, particularly black and Asian populations, when compared to historic emergency admissions across ICHNT in 2018-2019. When adjusted for age, Elixhauser comorbidity score and severity of disease on admission, higher odds of death for those from black ethnic background compared to whites were observed. |
| 19. Limitations | - 14% of our cohort were still hospitalized at the end of the reporting period, the most uncertain outcomes are likely to be amongst those who are currently still requiring mechanical ventilation - There are missing data for certain variables including ethnicity, BMI and novel laboratory biomarkers now systematically collected for patients with COVID-19, including D-dimer, BNP, troponin, ferritin and cortisol, which were excluded from regression analysis - We excluded patients who were SARS-CoV-2 RT-PCR-negative, those who might have had hospital-acquired SARS-CoV-2 infection - We did not quantify the impact of changes in clinical practice and experience over the time of our study |
| 20. Interpretation | The admission characteristics with the strongest association with increased odds of death were severe hypoxia, low platelet count, elevated bilirubin and a reduced eGFR. BAME groups were overrepresented in our study population, compared to historic data from ICHNT. After accounting for age, comorbidity profile and COVID-19 disease severity on admission, patients of black ethnic background appear to have increased odds of hospital mortality compared to those of white ethnicity |
| 21. Generalizability | Further work is needed to understand whether patterns observed in our study cohort are present on a national level and, if so, the sociodemographic and biologic factors underpinning it. Continuous monitoring of clinical data such as the one summarized in this study is paramount. Analyses are ongoing to test the out-of-sample validity of the clinical observations and investigations found to be significant in our study in predicting death outcomes from COVID-19 and whether other proposed biomarkers, such as D-Dimer and CRP are also useful predictors. |
| 22. Funding | MRC Centre for Global Infectious Disease Analysis  Abdul Latif Jameel Institute for Disease and Emergency Analytics  UK Medical Research Council  UK Department for International Development  The funders of this study had no role in study design, data collection, analysis, or interpretation, or writing of the report. The corresponding author had full access to all the data in the study and the final responsibility to submit for publication |

**References**

1. British Society of Thoracic Imaging. Thoracic Imaging in COVID-19 Infection. 2020: 28.

2. Elm E von, Altman DG, Egger M, Pocock SJ, Gøtzsche PC, Vandenbroucke JP. The Strengthening the Reporting of Observational Studies in Epidemiology (STROBE) statement: guidelines for reporting observational studies. Lancet (London, England) **2007**; :1453–57.
